# Transferrin Receptor 1 Regulates Thermogenic Capacity and Cell Fate in Brown/Beige Adipocytes

**DOI:** 10.1002/advs.201903366

**Published:** 2020-04-24

**Authors:** Jin Li, Xiaohan Pan, Guihua Pan, Zijun Song, Yao He, Susu Zhang, Xueru Ye, Xiang Yang, Enjun Xie, Xinhui Wang, Xudong Mai, Xiangju Yin, Biyao Tang, Xuan Shu, Pengyu Chen, Xiaoshuang Dai, Ye Tian, Liheng Yao, Mulan Han, Guohuan Xu, Huijie Zhang, Jia Sun, Hong Chen, Fudi Wang, Junxia Min, Liwei Xie

**Affiliations:** ^1^ The First Affiliated Hospital Institute of Translational Medicine School of Public Health Zhejiang University School of Medicine Hangzhou 310058 China; ^2^ Beijing Advanced Innovation Center for Food Nutrition and Human Health China Agricultural University Beijing 100193 China; ^3^ Department of Nutrition Precision Nutrition Innovation Center School of Public Health Zhengzhou University Zhengzhou 450001 China; ^4^ State Key Laboratory of Applied Microbiology Southern China Guangdong Provincial Key Laboratory of Microbial Culture Collection and Application Guangdong Open Laboratory of Applied Microbiology Guangdong Institute of Microbiology Guangdong Academy of Sciences Zhujiang Hospital Southern Medical University Guangzhou 510070 China; ^5^ Nanfang Hospital Southern Medical University Guangzhou 510515 China; ^6^ BGI Institute of Applied Agriculture BGI‐Shenzhen Shenzhen 518120 China

**Keywords:** beigeing, brown fat determination, HIF1α, thermogenesis, transferrin receptor 1

## Abstract

Iron homeostasis is essential for maintaining cellular function in a wide range of cell types. However, whether iron affects the thermogenic properties of adipocytes is currently unknown. Using integrative analyses of multi‐omics data, transferrin receptor 1 (Tfr1) is identified as a candidate for regulating thermogenesis in beige adipocytes. Furthermore, it is shown that mice lacking *Tfr1* specifically in adipocytes have impaired thermogenesis, increased insulin resistance, and low‐grade inflammation accompanied by iron deficiency and mitochondrial dysfunction. Mechanistically, the cold treatment in beige adipocytes selectively stabilizes hypoxia‐inducible factor 1‐alpha (HIF1α), upregulating the *Tfr1* gene, and thermogenic adipocyte‐specific *Hif1α* deletion reduces thermogenic gene expression in beige fat without altering core body temperature. Notably, *Tfr1* deficiency in interscapular brown adipose tissue (iBAT) leads to the transdifferentiation of brown preadipocytes into white adipocytes and muscle cells; in contrast, long‐term exposure to a low‐iron diet fails to phenocopy the transdifferentiation effect found in *Tfr1*‐deficient mice. Moreover, mice lacking transmembrane serine protease 6 (Tmprss6) develop iron deficiency in both inguinal white adipose tissue (iWAT) and iBAT, and have impaired cold‐induced beige adipocyte formation and brown fat thermogenesis. Taken together, these findings indicate that Tfr1 plays an essential role in thermogenic adipocytes via both iron‐dependent and iron‐independent mechanisms.

## Graphical abstract

A role of transferrin receptor 1 (Tfr1) in brown/beige adipocytes is reported. Ablation of Tfr1 in adipose tissue attenuates the thermogenic capacity and induces transdifferentiation of brown adipocytes via distinct mechanisms. The findings shed light on how Tfr1 and iron homeostasis affect thermogenesis, suggesting the Tfr1 and iron metabolic pathway as potential targets for preventing metabolic diseases.

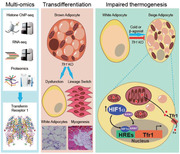

## 1 Introduction

Obesity occurs from an imbalance between energy intake and the body's consumption of energy, and excessive accumulation of fat contributes to the development of obesity. The body contains three types of adipose tissues, namely brown adipose tissue (BAT), white adipose tissue (WAT), and a form of WAT in which WAT cells can be converted to BAT‐like adipocytes via a process known as “beigeing”, resulting in beige (or “brite”) adipocytes. WAT is composed primarily of large unilocular lipid droplets that store triglycerides. In contrast, BAT is composed primarily of small multilocular lipid droplets that contain a large number of mitochondria, which burn systemic glucose and lipids in order to generate heat, a process known as thermogenesis. Beige adipose tissue is derived from WAT that has undergone beigeing due to environmental cold or other stimuli such as β3‐adrenergic signaling.^[^ ^1^ ^]^ During stimulation, the proteins EBF2 (early B cell factor‐2), EHMT1 (euchromatic histone‐lysine N‐methyltransferase 1), and PRDM16 (PR domain containing 16) are recruited to activate the expression of PGC1α (peroxisome proliferator‐activated receptor gamma coactivator 1‐alpha), followed by mitochondrial biosynthesis and the production of multilocular lipid droplets. Following stimulation, beige adipocytes are then converted back to WAT.^[^ ^2^, ^3^ ^]^ In mice, both brown and beige adipocytes underlie the adaptive non‐shivering thermogenic response and can regulate whole‐body energy metabolism, helping prevent obesity in animals exposed to a high‐fat diet (HFD).^[^ ^4^ ^]^ Thus, activating thermogenesis in brown and/or beige adipocytes has been considered as a promising therapeutic approach for preventing metabolic diseases such as type 2 diabetes (T2D), obesity, and cardiovascular disease.^[^ ^5^ ^]^

Iron plays an essential role in maintaining the balance between nutrients and energy metabolism, and several epidemiological studies have linked dysregulated iron homeostasis to obesity, T2D, and metabolic syndrome.^[^ ^6^, ^7^, ^8^ ^]^ The relationship between iron and white adipocytes has been studied intensively. For example, knocking down transferrin or chelating iron using deferoxamine (DFO) significantly inhibits adipogenesis and mitochondrial biosynthesis;^[^ ^9^ ^]^ in contrast, treating adipocytes with either iron or transferrin promotes lipolysis.^[^ ^10^ ^]^ Moreover, DFO has been reported to reduce adiposity and increase insulin sensitivity in *ob/ob* mice.^[^ ^11^ ^]^ Recently, Folgueras et al. reported that mice lacking transmembrane serine protease 6 (Tmprss6) are less susceptible to HFD‐induced obesity, suggesting that iron plays a regulatory role in adipose tissue.^[^ ^12^ ^]^ In addition, Wang et al. proposed that iron can accumulate in thermogenic adipocytes when exposed to cold.^[^ ^13^ ^]^ Finally, both animal experiments and clinical trials have provided evidence supporting the notion that nutritional iron deficiency impairs thermogenic capacity and lowers body temperature upon exposure to cold.^[^ ^14^, ^15^ ^]^ However, the role of iron homeostasis in thermogenic adipocytes particularly with respect to how iron affects thermogenesis is poorly understood.

Here, we performed integrative analyses of H3K9/14Ac‐ChIP‐seq, RNA‐seq, and iTRAQ proteomics profiling data and found that the membrane protein transferrin receptor 1 (Tfr1) is critical for the endocytosis of transferrin‐bound iron (TBI) in beige adipocytes. Although Tfr1 mediates iron uptake by internalizing TBI throughout the body, primarily in hepatocytes and erythrocytes,^[^ ^16^, ^17^ ^]^ its biological function in thermogenic adipose tissue has not been investigated. We found that Tfr1‐mediated iron uptake is essential for white adipocyte beigeing and the function of brown adipose tissue. Specifically, we found that mice lacking *Tfr1* in adipocytes have significantly impaired thermogenesis, together with iron deficiency and impaired mitochondrial function. In beige adipocytes, cold treatment selectively stabilized hypoxia‐inducible factor 1‐alpha (*Hif1α*), thereby upregulating its transcriptional target *Tfr1*. In contrast, loss of *Tfr1* in brown adipocytes drives the transdifferentiation of brown preadipocytes into white adipocytes and muscle cells, irrespective of iron status. Taken together, our results suggest that Tfr1 has a previously unrecognized role in the development and fate determination of brown/beige adipocytes.

## 2 Results

### 2.1 Multi‐Omics Profiling Reveals *Tfr1* as a Candidate Gene in Beige Adipocytes

**Figure 1:**
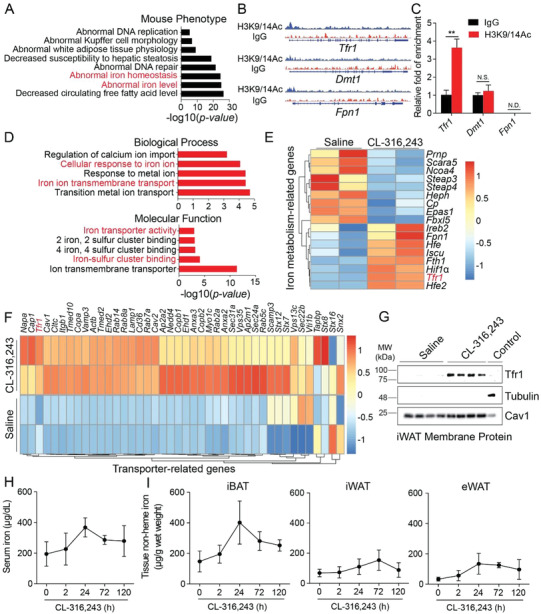
Tfr1‐mediated iron uptake plays a role in CL‐316,243‐induced beigeing of adipocytes in mice. A) Mouse phenotype analysis of H3K9/14Ac ChIP‐seq‐enriched fragment annotation. B) Traces of H3K9/14Ac ChIP‐seq fragment enrichment on the proximal promoter region of the indicated iron‐related genes. C) ChIP‐qPCR confirmation of H3K9/14Ac ChIP fragment enrichment of the indicated iron‐related genes. D) Gene ontology (GO: biological process and molecular function) analysis of differentially expressed genes. E) Heatmap of the iron metabolism‐related genes in adipocytes of mice treated with either saline or CL‐316,243. F) Heatmap of differentially expressed transporter‐related genes. G) Western blot analysis of iWAT membrane proteins showing increased Tfr1 expression in CL‐316,243‐treated mice. H,I) Time course of serum iron and iBAT, iWAT, and eWAT non‐heme iron levels in *C57BL/6J* mice following CL‐316,243 treatment (*n* = 6 mice/group), presented as mean ± SD, pooled from two independent experiments. **p < 0.01; N.S., not significant; and N.D., not detectable. Unpaired Student's *t*‐test was used for comparison between two groups. One‐way ANOVA with a Bonferroni post hoc analysis was used for comparison among multiple groups.

To generate an unbiased expression profile of the genes involved in the development of beige adipocytes, we used multi‐omics screening to identify potential gene candidates. First, we performed H3K9/14Ac ChIP‐seq in order to identify the active promoter in beige adipocytes using nuclei isolated from beige adipocytes in *C57BL/6J* mice treated for 5 days with the β3‐adrenergic agonist CL‐316,243 (Figure S1A, Supporting Information). We found that CL‐316,243 caused significant changes in genes expression of both iron level and iron homeostasis (**Figure** **1**A). Interestingly, H3K9/14Ac binding was increased in the promoter regions of several iron transport‐related genes, including *Tfr1*, *Dmt1*, and *Fpn1* (Figure 1B); H3K9/14Ac ChIP‐qPCR revealed that only the *Tfr1* proximal promoter was significantly enriched in H3K9/14Ac‐bound fragments in beige adipocytes (Figure 1C).

Next, we performed genome‐wide RNA‐seq in order to systematically profile the gene expression pattern in beige adipocytes in response to CL‐316,243. Our analysis revealed a total of 2675 differentially expressed genes in beige adipocytes, including 1690 upregulated genes and 985 downregulated genes (Figure S1B, Supporting Information). With respect to the upregulated genes, gene ontology (GO: biological process and molecular function) analysis showed that these differentially expressed genes were significantly enriched for iron transporter activity, iron ion transmembrane transport, and cellular response to iron ions (Figure 1D). Importantly, we found that iron metabolism related genes including *Tfr1* were significantly upregulated in beige adipocytes following CL‐316,243 treatment (Figure 1E).

Given that iron transmembrane transporter activity was the most significantly enriched molecular function (Figure 1D), we attempted to identify which membrane‐bound proteins are increased upon activation of beige adipocytes. We therefore isolated membrane proteins from *C57BL/6J* mice following injections of either CL‐316,243 (0.1 mg kg^−1^) or saline and performed proteomics analyses using the isobaric tag for absolute quantification (iTRAQ) approach. Our iTRAQ proteomics analysis revealed 473 upregulated membrane proteins (Figure S1C, Supporting Information), with *Tfr1* having significant increase in response to CL‐316,243 (Figure 1E,F). This finding was supported by isolating membrane proteins and performing western blot analysis, which showed that Tfr1 protein levels were increased in beige adipocytes in mice treated with CL‐316,243 (Figure 1G). Taken together, these multi‐omics analyses suggest that Tfr1 may play a fundamental role in inducing white adipocyte beigeing.

### 2.2 Tfr1‐Mediated Iron Uptake Is Increased During White Adipocyte Beigeing

In brown adipocytes, we found high levels of *Tfr1* mRNA and Tfr1 protein levels compared to both inguinal WAT (iWAT) and epididymal WAT (eWAT) adipocytes in *C57BL/6J* mice at steady state (Figure S1D,E, Supporting Information). To investigate the time course of Tfr1 expression during adipocyte thermogenesis, *C57BL/6J* mice were injected with CL‐316,243 (0.1 mg kg^−1^), and mRNA and protein levels were measured at 0, 2, 24, 72, and 120 h. We found that both *Tfr1* mRNA and protein levels gradually increased in iWAT adipocytes, which was followed by an upregulation of both PGC1α and Ucp1 (uncoupling protein 1) (Figure S1F,G, Supporting Information). In contrast, *Tfr1* expression was not significantly upregulated in iBAT adipocytes following CL‐316,243 injections, despite an upregulation of Ucp1 expression in these cells (Figure S1H,I, Supporting Information). Moreover, serum iron levels transiently increased after CL‐316,243 injection, reaching peak levels at 24 h (Figure 1H). This increase in serum iron levels was paralleled by an increase in tissue non‐heme iron in the adipose tissue of CL‐316,243‐injected mice, including iBAT, iWAT, and eWAT, with the largest increase measured in iBAT (Figure 1I).

### 2.3 Tfr1 Is Required for the Development of Beige and Brown Adipose Tissue

**Figure 2:**
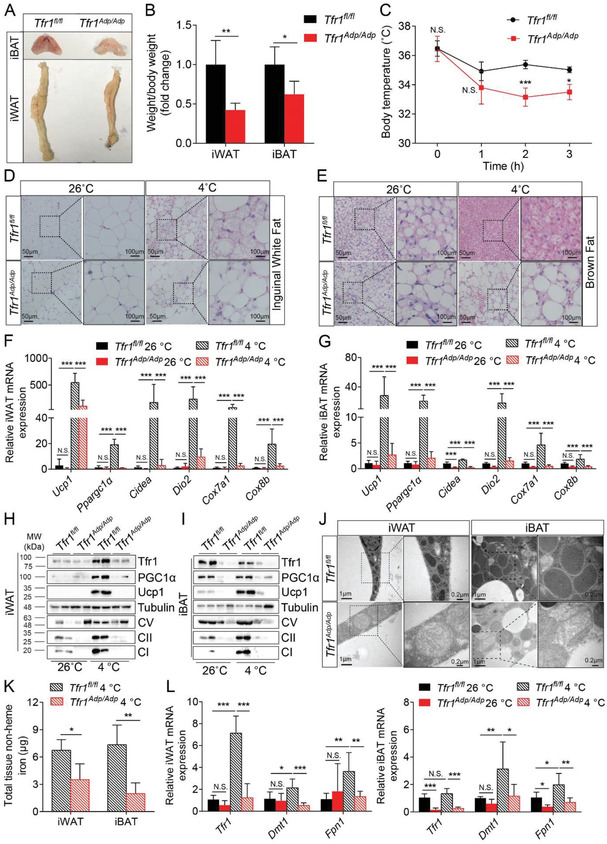
Adipocyte‐specific deletion of *Tfr1* reduces cold‐induced adipocyte thermogenesis. A) Representative images of iBAT and iWAT tissues from control (*Tfr1^fl/fl^*) and *Tfr1^Adp/Adp^* mice. B) Tissue weight of iBAT and iWAT in control (*Tfr1^fl/fl^*) *and Tfr1^Adp/Adp^* mice, expressed relative to the respective control (*n* = 4–5 mice per group), presented as mean ± SD, pooled from two independent experiments. C) Time course of rectal temperature of *Tfr1^fl/fl^* and *Tfr1^Adp/Adp^* mice at 4 °C (*n* = 4–5 mice per group), presented as mean ± SD, pooled from two independent experiments. D,E) Representative images of H&E‐stained iWAT and iBAT sections from *Tfr1^fl/fl^* and *Tfr1^Adp/Adp^* mice housed for 7 days at room temperature (26 °C) or 4 °C (*n* = 4–6 mice per group. F,G) qRT‐PCR analysis of thermogenesis‐related gene expression in iWAT and iBAT samples from *Tfr1^fl/fl^* and *Tfr1^Adp/Adp^* mice housed for 7 days at 26 or 4 °C (*n* = 4–6 mice per group), presented as mean ± SD, pooled from three independent experiments. H,I) Representative western blot images of iWAT and iBAT samples from *Tfr1^fl/fl^* and *Tfr1^Adp/Adp^* mice housed for 7 days at 26 or 4 °C (*n* = 4–6 mice per group). J) Representative transmission electron microscopy images of iWAT and iBAT samples from *Tfr1^fl/fl^* and *Tfr1^Adp/Adp^* mice. K) Total tissue non‐heme iron in iWAT and iBAT in *Tfr1^fl/fl^ and Tfr1^Adp/Adp^* mice housed for 7 days at 26 or 4 °C (*n* = 4–6 mice per group), presented as mean ± SD, pooled from three independent experiments. L) qRT‐PCR analysis of iron metabolism related gene expression in iWAT and iBAT from *Tfr1^fl/fl^* and *Tfr1^Adp/Adp^* mice housed for 7 days at 26 or 4 °C (*n* = 4–6 mice per group), presented as mean ± SD, pooled from three independent experiments. *p < 0.05, **p < 0.01, ***p < 0.001 and N.S., not significant. Unpaired Student's *t*‐test was used for comparison between two groups. One‐way ANOVA with a Bonferroni post hoc analysis was used for comparison among multiple groups.

Next, we examined the role of Tfr1 in thermogenic adipocytes by generating an adipocyte‐specific *Tfr1* knockout mouse (*Tfr1^Adp/Adp^*) mice by crossing *Tfr1^fl/fl^* with *Adipoq‐Cre* mice. Interestingly, we found significantly reduced amounts of both iWAT and iBAT in *Tfr1^Adp/Adp^* mice compared to control (*Tfr1^fl/fl^*) littermates (**Figure** **2**A,B), despite no significant difference in total body weight (Figure S2A, Supporting Information). In addition, we found that iBAT was visibly more whitish in color in *Tfr1^Adp/Adp^* mice compared to controls (Figure 2A). During prolonged exposure to cold, the body temperature of control mice first decreased slightly but then remained stable at approximately 35 °C; in contrast, the body temperature of *Tfr1^Adp/Adp^* mice decreased steadily from 37 °C to approximately 32 °C within 3 h (Figure 2C).

The ^18^fluoro‐2‐deoxyglucose positron emission tomography (^18^FDG‐PET) scanning has been widely used to determine the presence of iBAT in human body.^[^ ^18^ ^]^ Our micro‐PET/CT imaging results also revealed that the uptake of ^18^fluoro‐2‐deoxyglucose (^18^FDG) in iBAT was significantly reduced in *Tfr1^Adp/Adp^* mice compared to controls (Figure S2B, Supporting Information). Next, we investigated whether Tfr1 plays a role in the development of brown/beige adipocytes by exposing *Tfr1^Adp/Adp^* and control mice to cold (4 °C) and then staining iWAT and iBAT samples with hematoxylin and eosin (H&E). We found that cold exposure caused smaller multilocular lipid droplets in white adipocytes (i.e., beigeing in iWAT) in control mice, but not in *Tfr1^Adp/Adp^* mice (Figure 2D). Moreover, at room temperature (26 °C), iBAT in *Tfr1^Adp/Adp^* mice contained larger lipid droplets resembling classic white adipocytes compared to control iBAT, and cold exposure led to the development of small multilocular lipid droplets in control mice, but not in *Tfr1^Adp/Adp^* mice (Figure 2E). Notably, we also found that cold exposure significantly upregulated the expression of genes that encode critical thermogenic regulators (*Ucp1*, *Ppargc1α*, *Cidea*, *Dio2*, *Cox7a1*, and *Cox8b*) in both iWAT and iBAT in control mice, but had a substantially lower effect in *Tfr1^Adp/Adp^* mice (Figure 2F,G). Similar results were obtained with respect to the protein levels of Ucp1 and PGC1α measured in both iWAT (Figure 2H) and iBAT (Figure 2I), suggesting that *Tfr1^Adp/Adp^* mice have impaired brown/beige thermogenesis.

Since lipolysis is essential for thermogenesis by providing free fatty acids, especially for animals under external stimulation such as β‐agonist or cold exposure. We therefore measured the protein levels of HSL (hormone‐sensitive lipase), p‐HSL (phosphorylated HSL at S660 and S563) in iBAT and iWAT of *Tfr1^fl/fl^* and *Tfr1^Adp/Adp^* mice at RT and 4 °C, respectively. The results showed that upon cold exposure, HSL and p‐HSL protein levels were significantly decreased in both iBAT and iWAT of *Tfr1^Adp/Adp^* mice, suggesting reduced lipolysis in both iBAT and beige adipocytes (Figure S2C,D, Supporting Information). As a result, lower body temperature (Figure 2C) and larger lipid droplets (Figure 2D,E) were presented in *Tfr1^Adp/Adp^* mice compared with the control littermates. Taken together, adipocyte *Tfr1* deletion attenuates adipose tissue lipolysis, which could contribute to the observed impaired thermogenesis in *Tfr1^Adp/Adp^* mice.

In both iWAT and iBAT of *Tfr1^Adp/Adp^* mice, we also observed significantly reduced levels of mitochondrial complex I, II, and V proteins (Figure 2H,I), suggesting that loss of *Tfr1* in adipocytes causes impaired mitochondrial function. Consistent with this hypothesis, transmission electron microscopy revealed swollen mitochondria with irregular cristae in both iWAT and iBAT of *Tfr1^Adp/Adp^* mice, compared to normal mitochondria in control mice (Figure 2J). Next, we examined whether loss of *Tfr1* affects iron metabolism in adipose tissue by measuring both tissue iron content and the expression of iron transport‐related genes in iWAT and iBAT isolated from control and *Tfr1^Adp/Adp^* mice following cold exposure. As shown in Figure 2K, the total non‐heme iron in the iBAT and iWAT of *Tfr1^Adp/Adp^* mice was significantly reduced upon cold treatment. Notably, we found no difference in iron content in the iWAT between *Tfr1^fl/fl^* and *Tfr1^Adp/Adp^* upon cold exposure when we normalized the non‐heme iron to tissue weight (Figure S2E, Supporting Information), indicating no cell‐intrinsic defect. However, because the iWAT is considerably smaller (Figure 2B), when calculated for total tissue, the total iron is lower (Figure 2K). In addition, we also found reduced expression of iron transport‐related genes in both iWAT and iBAT of *Tfr1^Adp/Adp^* mice compared to control mice (Figure 2L). Similar to CL‐316,243 treatment, exposing control mice to cold significantly upregulated *Tfr1* expression in iWAT, but not in iBAT. Other genes that code for iron transporters, including *Dmt1* and *Fpn1*, were also significantly upregulated in iWAT and iBAT of control mice but not *Tfr1^Adp/Adp^* mice following cold exposure (Figure 2L). Taken together, these results suggest that Tfr1 plays a critical role in regulating the development of brown/beige adipocytes and in maintaining their thermogenic capacity.

### 2.4 Tfr1 Deficiency Increases HFD‐Induced Dyslipidemia, Insulin Resistance, and Inflammation

**Figure 3:**
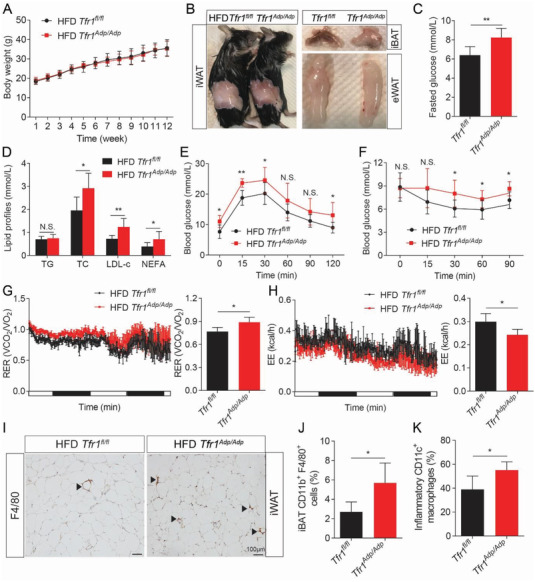
Mice with adipocyte‐specific *Tfr1* deficiency have increased HFD‐induced dyslipidemia, insulin resistance, and local inflammation. A) Starting at 6 weeks of age, male *Tfr1^fl/fl^* and *Tfr1^Adp/Adp^* mice were fed a high‐fat diet (HFD), and their body weight was measured each week (*n* = 7–8 mice per group), presented as mean ± SD, pooled from three independent experiments. B) HFD‐fed *Tfr1^fl/fl^* and *Tfr1^Adp/Adp^* mice were sacrificed at 14 weeks, and representative images of iWAT, eWAT, and iBAT are shown (*n* = 7–8 mice per group). C,D) Fasting glucose and plasma lipids were measured in HFD‐fed *Tfr1^fl/fl^* and *Tfr1^Adp/Adp^* mice (*n* = 7–8 mice per group), presented as mean ± SD, pooled from two independent experiments. E,F) The glucose tolerance test (GTT, E) and insulin tolerance test (ITT, F) were performed in HFD‐fed *Tfr1^fl/fl^* and *Tfr1^Adp/Adp^* mice (*n* = 7–8 mice per group), presented as mean ± SD, pooled from two independent experiments. G,H) Respiratory exchange rate (VO_2_/VCO_2_) and energy expenditure were monitored over a 48‐h period in HFD‐fed *Tfr1^fl/fl^* and *Tfr1^Adp/Adp^* mice (*n* = 4 mice per group), presented as mean ± SD; the white and black bars indicate when the lights were on and off, respectively. I) Representative images of iWAT sections immunostained for F4/80; arrowheads indicate F4/80^+^ cells. J,K) The percentage of CD11b^+^F4/80^+^ macrophages and pro‐inflammatory CD11c^+^ macrophages were measured in iBAT SVF using flow cytometry (*n* = 4 mice per group), presented as mean ± SD. *p < 0.05, **p < 0.01, and N.S., not significant. Unpaired Student's *t*‐test was used for comparison between two groups. One‐way ANOVA with a Bonferroni post‐hoc analysis was used for comparison among multiple groups.

Next, we examined the metabolic consequences of deleting *Tfr1* expression in adipocytes by feeding control and *Tfr1^Adp/Adp^* mice with HFD containing 60% fat for 12 weeks, thereby inducing obesity. We found no significant differences in body weight (**Figure** **3**A) or white adipose tissue mass including iWAT and eWAT (Figure 3B and Figure S3A, Supporting Information) between *Tfr1^Adp/Adp^* mice and controls. In contrast, we found that brown adipose tissue was completely transformed into white adipose tissue in HFD‐fed *Tfr1^Adp/Adp^* mice, but not in HFD‐fed controls (Figure 3B). In addition, HFD‐fed *Tfr1^Adp/Adp^* mice had significantly higher levels of fasting glucose and blood lipids including total cholesterol (TC), LDL‐cholesterol (LDL‐c), and non‐esterified fatty acids (NEFAs) compared to HFD‐fed controls (Figure 3C,D).

We also measured blood glucose levels during the glucose tolerance test (GTT) and insulin resistance test (ITT) in *Tfr1^Adp/Adp^* and control mice that were fed either a normal fat diet (Figure S3B,C, Supporting Information) or a high‐fat diet (Figure 3E,F). Interestingly, blood glucose levels were similar between *Tfr1^Adp/Adp^* and control mice maintained on a normal fat diet (Figure S3B,C, Supporting Information); in contrast, HFD‐fed *Tfr1^Adp/Adp^* mice had a significantly higher glucose response in both GTT and ITT (Figure 3E,F). Since there was no significant difference in percentage change of blood glucose in GTT (Figure S3D, Supporting Information), the higher overnight fasted glucose might be attributed to the impaired glucose tolerance in HFD‐fed *Tfr1^Adp/Adp^* mice compared with their controls.

To determine whether Tfr1 deficiency affects metabolic substrate preference and/or whole‐body fuel metabolism, we measured O_2_ consumption and CO_2_ production using metabolic cages. We found that HFD‐fed *Tfr1^Adp/Adp^* mice had a significantly higher respiratory exchange rate (Figure 3G) and significantly lower energy expenditure (Figure 3H) compared to HFD‐fed controls, indicating that HFD‐fed *Tfr1^Adp/Adp^* mice switched their preferred substrate from fat to carbohydrates and have impaired energy production. In addition, we observed no difference in the food intake between HFD‐fed *Tfr1^Adp/Adp^* mice and *Tfr1^fl/fl^* controls (Figure S3E, Supporting Information). However, we did observe a remarkable decreased amount of iWAT in normal diet‐fed *Tfr1^Adp/Adp^* mice compared to their controls (Figure 2A,B). Based on these observations, the compromised energy expenditure didn't result in increased body weight in HFD‐fed *Tfr1^Adp/Adp^* mice, probably due to the intrinsic effect of *Tfr1* deletion on white adipocytes.

Using immunohistochemistry and flow cytometry, we also found that significantly more macrophages infiltrated both the iWAT (Figure 3I) and iBAT (Figure 3J) of HFD‐fed *Tfr1^Adp/Adp^* mice. In addition, we found a higher percentage of inflammatory CD11c^+^ macrophages in the iBAT of HFD‐fed *Tfr1^Adp/Adp^* mice compared to HFD‐fed controls (Figure 3K; the gating strategy for flow cytometry analysis is shown in Figure S3F, Supporting Information). Notably, using transmission EM, we also found that the mitochondria were smaller and darker in iBAT of HFD‐fed *Tfr1^Adp/Adp^* mice compared to HFD‐fed controls (Figure S3G, Supporting Information). Additionally, we found only *Ppargc1α* and *Cidea* were significantly down‐regulated in iWAT of HFD‐fed *Tfr1^Adp/Adp^* mice compared to their respective HFD‐fed controls; whereas in iBAT, a wide range of thermogenic marker genes, including *Ucp1, Ppargc1α, Cidea, Cox7a1*, and *Cox8b* were significantly down‐regulated in HFD‐fed *Tfr1^Adp/Adp^* mice (Figure S3H, Supporting Information). Taken together, these data support the notion that Tfr1 in adipocytes plays an essential role in maintaining whole‐body energy expenditure.

### 2.5 Tfr1 Is Regulated by HIF1α in Beige Adipocytes

**Figure 4:**
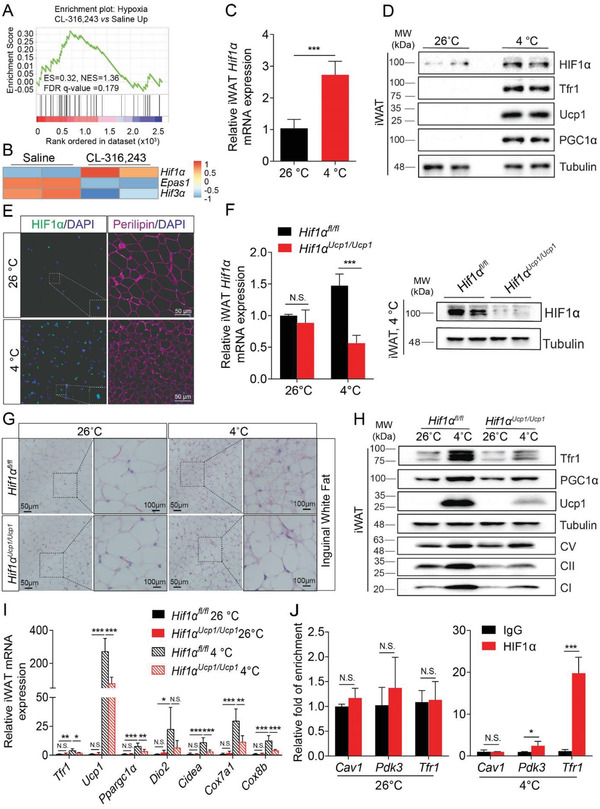
Nuclear HIF1α transactivates *Tfr1* expression in beige adipocytes. A) GSEA analysis of upregulated pathways in response to hypoxia. B) Heatmap of hypoxia‐inducible factor (HIF)‐related gene expression in beige adipocytes. C) qRT‐PCR analysis of *Hif1α* expression in white adipocytes in *C57BL/6J* mice housed at 26 or 4 °C for 7 days (*n* = 6 mice per group), presented as mean ± SD, pooled from two independent experiments. D) Representative western blot analysis of HIF1α, Tfr1, Ucp1, PGC1α, and Tubulin in iWAT from *C57BL/6J* mice housed at or 4 °C for 7 days (*n* = 6 mice per group). E) Representative images of iWAT sections obtained from *C57BL/6J* mice housed at 26 or 4 °C for 7 days and immunostained for HIF1α and Perilipin (*n* = 6 mice per group). F) qRT‐PCR and western blot analysis of *Hif1α* expression in iWAT in *Hif1α^fl/fl^* and *Hif1α^Ucp1Ucp1^* mice housed at 26 or 4 °C for 7 days (*n* = 4 mice per group), presented as mean ± SD, pooled from two independent experiments. G) Representative images of H&E‐stained iWAT sections from *Hif1α^fl/fl^* and *Hif1α^Ucp1Ucp1^* mice housed at 26 or 4 °C for 7 days (*n* = 4 mice per group). H) Representative western blot analysis of Tfr1, PGC1α, Ucp1, tubulin, and mitochondrial complexes I, II, and V in iWAT samples from *Hif1α^fl/fl^* and *Hif1α^Ucp1Ucp1^* mice housed at 26 or 4 °C for 7 days (*n* = 4 mice per group). I) qRT‐PCR analysis of the indicated adipocyte thermogenesis‐related genes in iWAT of *Hif1α^fl/fl^* and *Hif1α^Ucp1Ucp1^* mice housed at 26 or 4 °C for 7 days (*n* = 4 mice per group), presented as mean ± SD, pooled from two independent experiments. J) HIF1α‐ChIP‐qPCR showing the fold enrichment at the *Cav1*, *Pdk3*, and *Tfr1* promoters in beige adipocytes isolated from *GFP^Ucp1/Ucp1^* mice housed at 26 or 4 °C for 7 days (*n* = 6), presented as mean ± SD. *p < 0.05, **p < 0.01, ***p < 0.001, and N.S., not significant. Unpaired Student's *t*‐test was used for comparison between two groups. One‐way ANOVA with a Bonferroni post‐hoc analysis was used for comparison among multiple groups.

Next, we examined how *Tfr1* expression is regulated during beigeing by analyzing RNA‐seq data derived from iWAT samples obtained from *C57BL/6J* mice treated with CL‐316,243 or saline. A previous study demonstrated that cold treatment‐induced beige adipocyte‐specific hypoxia was detected by using the commercialized Hypoxyprobe‐1.^[^ ^19^ ^]^ Consistently, our Gene Set Enrichment Analysis (GSEA) revealed that the expression of hypoxia‐related genes was differentially regulated in the CL‐316,243‐treated group (**Figure** **4**A). Among these genes, *Hif1α* was significantly upregulated, whereas *Hif2α* (also known as *Epas1*) and *Hif3α* were both downregulated in the CL‐316,243‐treated group (Figure 4B). Moreover, we found that HIF1α, Tfr1, Ucp1, and PGC1α protein levels were upregulated in the iWAT of *C57BL/6J* mice following exposure to cold (Figure 4C,D, and Figure S4A, Supporting Information). Interestingly, immunostaining revealed that HIF1α protein accumulated in the nuclei of iWAT adipocytes in mice exposed to cold (Figure 4E) and in mice treated with CL‐316,243 (Figure S4B, Supporting Information), suggesting that HIF1α activation may play a role in the transformation of beige adipocytes.

To further investigate the physiological role of HIF1α in brown and beige adipocytes, we crossed *Hif1α^fl/fl^* mice with *Ucp1‐Cre* mice to generate offsprings that lack *Hif1α* specifically in adipocytes with Ucp1 expression (referred to hereafter as *Hif1α^Ucp1/Ucp1^* mice). Exposing *Hif1α^Ucp1/Ucp1^* mice to cold (4 °C) for 7 days caused a significant decrease in *Hif1α* mRNA and decreased HIF1α protein levels to <10% of control levels (Figure 4F). Moreover, loss of *Hif1α* in iWAT completely reversed cold‐induced beigeing (Figure 4G). Consistent with reduced HIF1α expression, the increase in mitochondrial complex proteins seen in the iWAT of cold‐exposed control mice was significantly reduced in cold‐exposed *Hif1α^Ucp1/Ucp1^* mice (Figure 4H), as was the expression of genes related to thermogenesis and mitochondrial biogenesis, including *Ucp1*, *Ppargc1α*, *Dio2*, *Cidea*, *Cox7a1*, and *Cox8b* (Figure 4I).

In contrast, HIF1α protein was virtually undetectable in iBAT, regardless of genotype and external temperature (Figure S4C, Supporting Information), suggesting that HIF1α does not appear to play a role in brown adipocytes. Moreover, both western blot analysis (Figure S4C, Supporting Information) and H&E staining (Figure S4D, Supporting Information) showed that iBAT in cold‐exposed *Hif1α^Ucp1/Ucp1^* mice contains normal‐size lipid droplets and normal expression of thermogenesis and mitochondrial complex proteins. In iBAT, the iron‐related gene expression including *Tfr1*, *Dmt1*, and *Fpn1* remain unchanged between *Hif1a^fl/fl^* and *Hif1a^Ucp1/Ucp1^* mice either at RT or 4 °C, respectively (Figure S4E, Supporting Information). Interestingly, we found that loss of *Hif1α* in thermogenic adipocytes did not affect the core body temperature in cold‐exposed *Hif1α^Ucp1/Ucp1^* mice compared to that of the control littermates (Figure S4F, Supporting Information), suggesting that HIF1α is likely involved, but not essential per se, to support adaptive thermogenesis.

Notably, cold‐induced loss of *Hif1α* also reduced the induction of *Tfr1* expression in iWAT (Figure 4H,I), which suggests that the *Tfr1* gene may be transcriptionally regulated by HIF1α. To test this hypothesis, we performed HIF1α ChIP‐qPCR analysis and found that HIF1α is indeed enriched at the *Tfr1* promoter during beigeing (i.e., upon cold exposure); as a positive control, we also found that HIF1α was enriched in the *Pdk3* promoter (Figure 4J).^[^ ^20^ ^]^ Taken together, these results indicate that HIF1α‐Tfr1 signaling cascade plays a critical role in the development of beige adipocytes.

### 2.6 Loss of *Tfr1* in Adipocytes Promotes the Transdifferentiation of Brown Adipocytes into Muscle‐Like Cells

**Figure 5:**
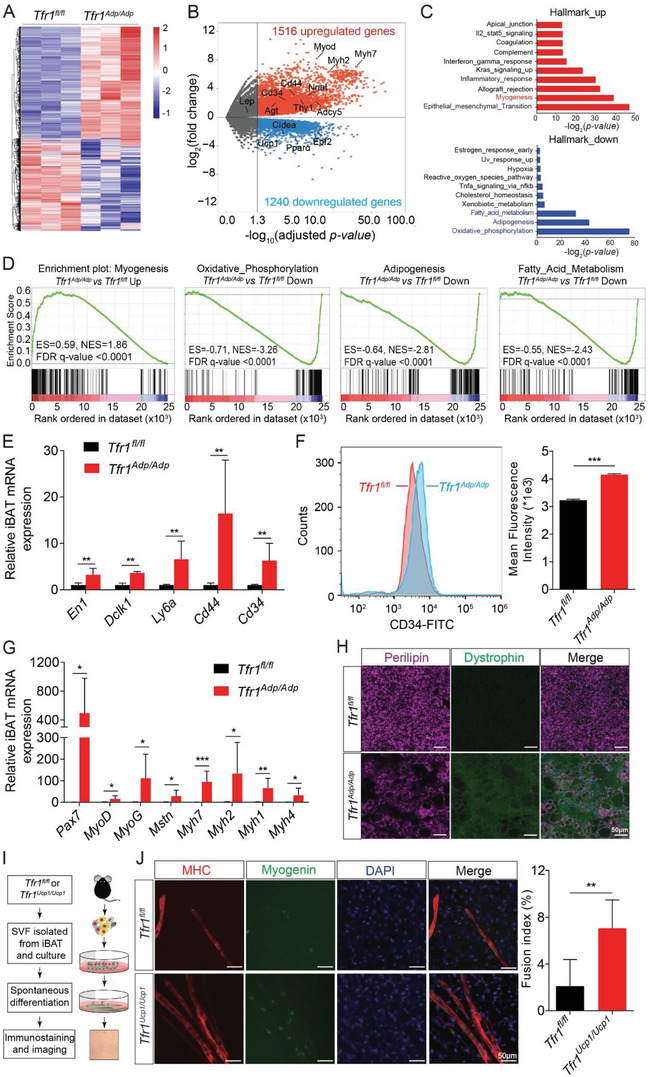
Adipocyte‐specific loss of Tfr1 causes the transdifferentiation of brown preadipocytes into muscle‐like cells. A) mRNA expression profile of iBAT in *Tfr1^fl/fl^* and *Tfr1^Adp/Adp^* mice (*n* = 3 mice per group). B) Volcano plot of differentially expressed genes in iBAT in *Tfr1^fl/fl^* and *Tfr1^Adp/Adp^* mice. Biomarkers associated with skeletal muscle, stem cells, thermogenesis, and white adipocytes are indicated. C) GSEA (Hallmark) analysis of differentially expressed genes. D) GSEA analysis of the most strongly upregulated (myogenesis) and downregulated (oxidative phosphorylation, adipogenesis, and fatty acid metabolism) pathways. E) qRT‐PCR analysis of stem cell‐related genes in iBAT of *Tfr1^fl/fl^* and *Tfr1^Adp/Adp^* mice (*n* = 6 mice per group), presented as mean ± SD, pooled from 3 independent experiments. F) Flow cytometry analysis of the stem cell marker CD34 in SVF samples obtained from the iBAT of *Tfr1^fl/fl^* and *Tfr1^Adp/Adp^* mice (*n* = 3 mice per group), presented as mean ± SD. G) qRT‐PCR analysis of myogenesis‐related genes in iBAT of *Tfr1^fl/fl^* and *Tfr1^Adp/Adp^* mice (*n* = 4–6 mice per group), presented as mean ± SD, pooled from two independent experiments. H) Representative images of iBAT sections from *Tfr1^fl/fl^* and *Tfr1^Adp/Adp^* mice immunostained for perilipin and dystrophin (*n* = 4–6 mice per group). I) Schematic cartoon depicting the experimental design for preparing and analyzing primary brown adipocytes from *Tfr1^fl/fl^* and *Tfr^Ucp1/Ucp1^* mice. J) Representative images and summary of fusion index of primary brown adipocytes immunostained for myosin heavy chain (MHC) and myogenin; the nuclei were counterstained with DAPI (*n* = 6 mice per group), presented as mean ± SD, pooled from three independent experiments. *p < 0.05, **p < 0.01, and ***p < 0.001. Unpaired Student's *t*‐test was used for comparison between two groups.

Next, we attempted to identify genes and/or pathways that contribute to the developmental defect in iBAT in the absence of Tfr1. We therefore analyzed the gene expression profiles of iBAT samples obtained from control (*Tfr1^fl/fl^*) and *Tfr1^Adp/Adp^* mice and found a total of 3796 genes that were differentially expressed, with 1516 upregulated genes and 1240 downregulated genes (**Figure** **5**A). As expected, we found that the brown fat marker genes *Ucp1*, *Pparα*, *Cidea*, and *Ebf2* were significantly downregulated in *Tfr1^Adp/Adp^* mice, whereas gene markers of white adipocytes such as *Agt*, *Nnat*, and *Adcy5* were significantly upregulated (Figure 5B); the upregulation of white adipocyte‐related genes were confirmed using quantitative real‐time PCR (qRT‐PCR) (Figure S5A, Supporting Information). Notably, a typical white adipose marker gene leptin (*Lep*) displayed no significant change at RT, but significantly up‐regulated in *Tfr1^Adp/Adp^* mice under cold stress (Figure S5A, Supporting Information). In addition, GSEA analyses revealed that several upregulated gene sets are involved in myogenesis, whereas many downregulated genes are associated with oxidative phosphorylation, adipogenesis, and fatty acid metabolism (Figure 5C,D and Figure S5B–E, Supporting Information). Similar to previous findings in *Tfr1*‐deficient intestine epithelial cells,^[^ ^21^ ^]^ we found that several stem cell marker genes, including *En1*, *Dclk1*, *Ly6a*, *Cd34*, and *Cd44*, were also significantly upregulated in iBAT of *Tfr1^Adp/Adp^* mice compared to control mice (Figure 5B,E); the upregulation of CD34 expression was confirmed at the protein level using flow cytometry (Figure 5F). Interestingly, we also found that the myogenic signature genes *Pax7*, *MyoD*, *MyoG*, *Mstn*, *Myh7*, *Myh2*, *Myh1*, and *Myh4* were also significantly upregulated (Figure 5B,G). In addition, co‐immunostaining iBAT samples from *Tfr1^Adp/Adp^* mice showed decreased expression of perilipin (a lipid droplet‐specific marker) and increased expression of the myofiber‐specific marker dystrophin (Figure 5H), suggesting that reprograming of brown adipocyte progenitor cells may underlie the shift to a muscle cell‐like phenotype.

To exclude the possible effect of other endocrine cells such as white adipocytes and hepatocytes, we generated mice lacking *Tfr1* specifically in thermogenic adipocytes (referred to hereafter as *Tfr1^Ucp1/Ucp1^* mice) by crossing *Tfr1^fl/fl^* mice with the *Ucp1‐Cre* mouse line. We confirmed that Tfr1 expression was successfully deleted in iBAT (Figure S6A,B, Supporting Information) but was still expressed in the liver and iWAT (Figure S6C, Supporting Information) of *Tfr1^Ucp1/Ucp1^* mice. Furthermore, we found that the myogenesis‐related proteins Pax7, MyoD, and myogenin were upregulated in iBAT of *Tfr1^Ucp1/Ucp1^* mice compared to control littermates (*Tfr1^fl/fl^*) (Figure S6A,B, Supporting Information).

Next, we isolated the stromal vascular fraction (SVF, a population enriched for precursor cells) from the iBAT of both *Tfr1^Ucp1/Ucp1^* and control mice in order to examine the role of Tfr1 in the lineage switch from brown preadipocytes to muscle precursor cells (Figure 5I). Using immunofluorescence, we found that *Tfr1^Ucp1/Ucp1^* brown preadipocytes spontaneously differentiate into myotubes with high expression of myosin heavy chain (MHC) and myogenin (Figure 5J). In addition, we found that SVF in *Tfr1^Ucp1/Ucp1^* mice has a higher potential to fuse to develop into myotube, demonstrated by higher fusion index compared to controls (approximately 7.1% vs 2.1%, respectively), supporting the notion that Tfr1 plays a role in lineage determination.

### 2.7 Iron Deficiency Impairs Thermogenesis but Does Not Affect the Transdifferentiation of Brown Adipocytes

**Figure 6:**
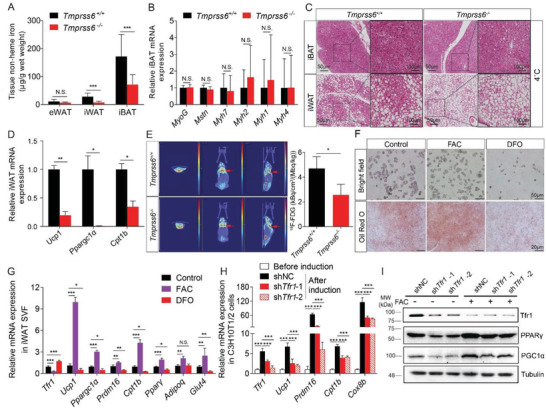
Iron deficiency affects thermogenic adipocytes. A) Non‐heme iron was measured in eWAT, iWAT, and iBAT of control (*Tmprss6^+/+^*) and *Tmprss6^−/−^* mice (*n* = 6 mice per group). presented as mean ± SD, pooled from two independent experiments. B) qRT‐PCR analysis of the indicated myogenesis genes in iBAT of *Tmprss6^+/+^* and *Tmprss6^−/−^* mice (*n* = 6 mice per group), presented as mean ± SD, pooled from two independent experiments. C) Representative images of H&E‐stained iBAT and iWAT in *Tmprss6^+/+^* and *Tmprss6^−/−^* mice housed for 7 days at 4 °C (*n* = 3 mice per group), presented as mean ± SD, from two independent experiments. D) qRT‐PCR analysis of *Ucp1*, *Ppargc1α*, *Cpt1b* mRNA in iWAT and iBAT of *Tmprss6^+/+^* and *Tmprss6^−/−^* mice housed for 7 days at 4 °C (*n* = 3 mice/group), presented as mean ± SD, from two independent experiments. E) Representative ^18^F‐FDG micro‐PET/CT images of *Tmprss6^+/+^* and *Tmprss6^−/−^* mice after single dose of CL‐316,243 (i.p. 1 mg kg^−1^) treatment for 1 h. The quantitative summary of FDG uptake is shown at the right (*n* = 3 mice per group). F) Representative bright field images and Oil Red O stained SVF after differentiation for 6 days in DMSO (control), 100 µm ferric ammonium citrate (FAC), or 5 µm desferrioxamine (DFO). G) qRT‐PCR analysis of *Tfr1*, *Ucp1*, *Ppargc1α*, *Prdm16*, *Cpt1b*, *Pparγ*, *Adiponectin*, and *Glut4* mRNA in iWAT SVF after differentiation for 6 days in DMSO, 100 µm FAC, or 5 µm DFO (*n* = 4 per group), presented as mean ± SD, pooled from two independent experiments. H) qRT‐PCR analysis of *Tfr1*, *Ppargc1α*, *Prdm16*, *Cpt1b*, and *Cox8b* mRNA in C3H10T1/2 cells stably expressing a non‐targeted shRNA (shNC) or two different *Tfr1* shRNA constructs (*n* = 4 per group), presented as mean ± SD, pooled from two independent experiments. I) Representative western blot analysis of Tfr1, PGC1α, and Pparγ proteins in C3H10T1/2 cells stably expressing shNC or *Tfr1* shRNAs treated either with or without 100 µm FAC (*n* = 3 per group), presented as mean ± SD, from two independent experiments. *p < 0.05, **p < 0.01, ***p < 0.001 and N.S., not significant. Unpaired Student's *t*‐test was used for comparison between two groups. One‐way ANOVA with a Bonferroni post hoc analysis was used for comparison among multiple groups.

Given that *Tfr1^Adp/Adp^* mice have significantly lower iron levels in both iWAT and iBAT, we next tested whether iron deficiency plays a causal role in the phenotypes observed in *Tfr1^Adp/Adp^* mice using a genetic mouse model of iron deficiency (*Tmprss6* ^−/−^ mice),^[^ ^22^ ^]^ We first confirmed that *Tmprss6* ^−/−^ mice lack hepcidin repressor matriptase‐2 (encoded by the *Tmprss6* gene) and have both increased hepcidin expression (Figure S7A, Supporting Information) and reduced iron levels (**Figure** **6**A) compared to control mice. Moreover, consistent with previous reports,^[^ ^22^ ^]^ the lipid droplets in iBAT were significantly smaller in *Tmprss6^−/−^* mice compared to controls (Figure S7B, Supporting Information). Surprisingly, however, expression of the myogenesis marker genes *MyoG*, *Mstn*, *Myh7*, *Myh2*, *Myh1*, and *Myh4* was similar between *Tmprss6^−/−^* mice and controls (Figure 6B). Upon cold exposure (4 °C), the *Tmprss6^−/−^* mice had significantly larger lipid droplets (Figure 6C) and down‐regulated thermogenic genes such as *Ucp1*, *Ppargc1α*, and *Cpt1b* in iWAT compared to control littermates (Figure 6D), indicating that white adipocyte beigeing is impaired in *Tmprss6^−/−^* mice. Although we found no significant difference in the size of lipid droplets and in the expression of *Ucp1* and *Cpt1b* in iBAT between cold‐exposed *Tmprss6^−/−^* mice and controls (Figure 6C and Figure S7C, Supporting Information). The uptake of ^18^FDG in iBAT was significantly reduced in CL‐316,243‐treated *Tmprss6^−/−^* mice compared to their controls (Figure 6E), suggesting impaired thermogenesis in iBAT of *Tmprss6^−/−^* mice.

To investigate whether systemic iron deficiency affects fetal iBAT development, we fed pregnant *C57BL/6J* dams either a low‐iron diet (LID) containing 3 ppm iron or a normal‐iron diet (NID) containing 33 ppm iron during pregnancy and through the lactation period until the offspring reached 3 weeks of age. We found that offspring exposed to the LID had significantly retarded growth compared to offspring that were exposed to the NID. Interestingly, at 3 weeks of age the heart, liver, spleen, and kidneys were smaller in LID‐exposed offspring, and the iBAT was also smaller and paler compared to NID‐exposed offspring. The iBAT gradually developed the similar size but still paler than iBAT in NID‐fed mice by 6 weeks of age (Figure S7D, Supporting Information). Consistent with phenotypes observed in *Tmprss6^−/−^* mice, the iron content was also significantly lower (Figure S7E, Supporting Information), concurrently with the up‐regulation of *Tfr1* mRNA expression (Figure S7F, Supporting Information), in the iBAT of prenatal LID‐exposed offspring. Noteworthy, in those LID‐exposed offspring, the thermogenic genes *Ucp1* and *Cpt1b* in the iBAT were slightly but significantly down‐regulated, as well as the expression of the myogenic signature genes, including *Myh7*, *Myh2*, *Myh1*, and *Myh4* but not *MyoD*, was also significantly down‐regulated (Figure S7F, Supporting Information). These findings suggest that exposure to a low‐iron diet starting at conception does not cause the transdifferentiation of brown preadipocytes into muscle cells.

Next, we tested the role of iron in adipocyte differentiation using an in vitro SVF culture system with Oil Red O to stain lipids. We found that treating SVF cells with ferric ammonium citrate (FAC) accelerated adipocyte differentiation, leading to increased accumulation of lipid droplets; in contrast, treating cells with the iron chelator DFO significantly reduced adipocyte differentiation (Figure 6F). In addition, FAC treatment significantly upregulated the thermogenic marker genes *Ucp1*, *Ppargc1α*, *Prdm16*, and *Cpt1b*, as well as the adipocyte markers *Pparγ*, *Adiponectin*, and *Glut4*; in contrast, DFO treatment significantly downregulated these genes (Figure 6G). Moreover, consistent with its role as an iron‐responsive gene, *Tfr1* expression was significantly downregulated in FAC‐treated cells and upregulated in DFO‐treated cells (Figure 6G).

Finally, to examine whether loss of Tfr1 is sufficient to prevent brown adipocyte differentiation in vitro, we used RNAi to knockdown *Tfr1* expression in the mouse embryonic fibroblast cell line C3H10T1/2. Compared with cells expressing a non‐targeted shRNA (shNC), cells expressing two independent shRNA constructs had significantly reduced expression of the thermogenic markers *Ppargc1α*, *Prdm16*, *Cpt1b*, and *Cox8b* (Figure 6H). Moreover, FAC partially rescued the effects of *Tfr1* shRNA on the differentiation of C3H10T1/2 cells into brown adipocytes, as PPARγ and PGC1α protein levels were similar to cells expressing the control shRNA (Figure 6I). Taken together, these data suggest that iron is essential for beige adipocyte formation but is not required for the lineage determination of brown adipocytes.

## 3 Discussion

The role of iron homeostasis in adipose tissue particularly in beige and brown adipocytes is poorly understood. Here, we identified and functionally characterized the essential role that Tfr1 plays in regulating the development of beige and brown adipocytes in vivo. We found that adipocyte‐specific deletion of *Tfr1* in mice leads to cold intolerance and impaired thermogenesis. In addition, adipocyte‐specific *Tfr1*‐deficient mice have dysregulated lipid metabolism and develop insulin resistance when challenged with a high‐fat diet. Mechanistically, we found that HIF1α transcriptionally regulates *Tfr1* expression during beigeing. We demonstrated that Tfr1 functions as a major player not only in the development of brown and beige adipocytes but also in thermogenesis. Further, we showed regulatory role of Tfr1 in thermogenesis is dependent on iron content in adipose tissues, whereas the role of Tfr1 in brown adipocyte lineage commitment is independent of iron status. Based on these observations, we conclude that Tfr1 may serve as a regulatory mechanism during thermogenic adipocyte function and fate determination.

It is well known that iron homeostasis is essential for maintaining cell growth and function.^[^ ^23^ ^]^ In nearly all eukaryotes, iron is delivered throughout the body by serum transferrin (Tf), a glycoprotein that binds iron and transports it into cells via Tfr‐mediated endocytosis. Thus, the membrane‐bound Tf‐Fe^3+^/Tfr1 complex is the major route of cellular iron uptake. However, whether Tfr1 plays a role in thermogenic adipocytes is currently unknown. Using unbiased integrative analyses of ChIP‐seq, RNA‐seq, and iTRAQ quantitative proteomics profiling data from mice treated with CL‐316,243, we found that Tfr1 was a leading candidate for further study. To overcome embryonic lethality associated with global *Tfr1* deletion due to severe iron deficiency anemia,^[^ ^17^ ^]^ we generated adipocyte‐specific *Tfr1* knockout (*Tfr1^Adp/Adp^*) mice and found that these mice do not develop subcutaneous beige fat when exposed to extreme cold (4 °C). Phenotypically, *Tfr1^Adp/Adp^* mice have iron deficiency, cold intolerance, impaired beige and brown adipocyte function, and impaired mitochondrial function.

Iron deficiency has been associated with mitochondrial dysfunction due to the role that iron plays in the biogenesis of mitochondrial Fe‐S clusters and electron transport chain subunits.^[^ ^24^ ^]^ Given that both beige and brown adipocytes contain large numbers of mitochondria for energy expenditure and thermogenesis, it is reasonable to speculate that the development of beige/brown adipocytes requires Tfr1 in order to take in sufficient amounts of iron to maintain normal mitochondrial function. Recently, Blankenhaus et al. reported that loss of the iron storage protein ferritin (Fth) impairs thermogenesis in iBAT, possibly due to a dysregulation of systemic iron homeostasis.^[^ ^25^ ^]^ In our study, genetic iron deficiency mice (*Tmprss6* null mice) had a detrimental effect on both cold‐induced beige adipocyte differentiation or CL‐316,243‐activated brown adipocytes.

In adipose tissue of lean mice, most of the macrophages have been identified as anti‐inflammatory M2‐like macrophages. By contrast, the majority of the macrophages in obese adipose tissue consists of activated pro‐inflammatory M1‐like macrophages.^[^ ^26^ ^]^ In our study, *Tfr1* deletion exacerbated HFD‐induced polarized pro‐inflammatory macrophage infiltration in adipose tissue, which may in turn significantly increase the secretion of pro‐inflammatory cytokines and lead to systemic insulin resistance.^[^ ^27^, ^28^ ^]^ Similarly, a recent study reported that these M2‐like macrophages in lean adipose tissue had more intracellular iron storage, whereas in obese adipose tissue, those M1‐like macrophages had impaired ability of handling iron recycling.^[^ ^29^ ^]^ Therefore, the significance of increased M1‐like macrophages (inflammatory CD11c^+^ macrophages) in *Tfr1^Adp/Adp^* mice might aggravate HFD‐induced insulin resistance and dysregulation of iron homeostasis in adipose tissue.

External factors such as exposure to cold or treatment with β‐adrenergic agonists stimulate adipocyte thermogenesis and the transformation of beige adipocytes, followed by an upregulation of *Tfr1* expression. It is well known that oxygen is required for maintaining adipocyte function.^[^ ^19^ ^]^ Low tissue oxygen leads to stabilization of hypoxia‐inducible transcription factors, including HIF1α, HIF2α, and HIF3α, which may preferentially regulate distinct aspects of adipocyte physiology and function such as glucose metabolism, angiogenesis, and adipose tissue function.^[^ ^30^ ^]^ Previous studies demonstrated that cold exposure induces tissue hypoxia in both brown and beige adipocytes, followed by upregulation of *Hif2α* mRNA expression only;^[^ ^19^, ^31^ ^]^ however, the physiological function of hypoxia inducible factors (HIFs) in thermogenic adipocytes development and function in vivo remains unclear. Here, we found that HIF1α was stabilized in beige adipocytes induced by cold exposure or β3‐adrenergic agonists, but not in iBAT either at room temperature or upon cold exposure. We also found that HIF1α was likely involved in beige adipocyte differentiation but not essential to support adaptive thermogenesis in BAT by mechanism of transcriptionally promoting *Tfr1* expression, which is consistent with the previous report that HIF1α binds to the *Tfr1* promoter in hepatocytes.^[^ ^32^ ^]^ Here, we show that cold exposure stabilizes HIF1α protein in beige adipocytes, directly activating *Tfr1* expression and subsequently promoting iron uptake. As previously reported, this observed phenomena could be mediated by cold exposure‐induced local tissue hypoxia.^[^ ^19^ ^]^ However, we could not exclude other potential involvement of HIF1α stabilization pathways, such as cellular redox changes, tissue repair pathways, and NFκB signaling pathways.^[^ ^33^, ^34^ ^]^

Intrascapular brown fat develops early in embryogenesis and originates from progenitor cells in the central dermomyotome, which specifically expresses the homeobox gene engrailed 1 (*En1*), myogenic factor 5 (*Myf5*), and paired box 7 (*Pax7*). The progenitor population in the central dermomyotome can also differentiate into white adipocytes, muscles, and dorsal dermis;^[^ ^35^ ^]^ thus, this population could undergo cell fate commitment to become brown preadipocytes, and then develop into mature brown adipocytes. To the best of our knowledge, this is the first report of a developmental defect in iBAT in adipose‐specific *Tfr1*‐deficient mice. Interestingly, our RNA‐seq analysis revealed that the strongest downregulated genes are involved in oxidative phosphorylation, adipogenesis, and fatty acid metabolism, suggesting that mitochondrial energy expenditure and lipid metabolism are all attenuated in iBAT of *Tfr1^Adp/Adp^* mice. On the other hand, we found robust upregulation of myogenesis genes, which indicates that the loss of *Tfr1* results in the reprogramming of brown preadipocytes into muscle cells. The switch from brown adipocytes to muscle cells has been linked to Ebf2,^[^ ^3^ ^]^ Prdm16,^[^ ^2^ ^]^ EHMT1,^[^ ^3^ ^]^ zinc finger protein 516 (ZFP516),^[^ ^4^ ^]^ and the non‐coding RNA microRNA‐133.^[^ ^36^ ^]^

Prdm16 is particularly interesting since it has been defined as a well‐known transcription factor in controlling the cell fate of brown fat. It is reported that overexpression of *Prdm16* inhibits myogenic differentiation in C2C12 myoblasts;^[^ ^2^ ^]^ whereas deletion of *Prdm16* leads to elevated expression of muscle‐related genes in Myf5‐expressing precursors.^[^ ^37^ ^]^ However, the most recent finding in mice challenges the notion. The *Prdm16* knockout mice showed no effect on the development of embryonic BAT but rather resulted in impaired BAT thermogenesis.^[^ ^37^ ^]^ Based on our in vitro data, either iron chelation in iWAT SVF cells (Figure 6G) or silencing of *Tfr1* in C3H10T1/2 cells (Figure 6H) reduced the expression of *Prdm16*, which suggests that Tfr1 might modulate *Prdm16* expression in adipose tissue.

In addition to iron acquisition, non‐canonical function of Tfr1 has been previously reported.^[^ ^21^, ^38^ ^]^ For example, Tfr1 could interact with HFE to modulate hepcidin expression in the liver.^[^ ^38^ ^]^ Whereas in intestinal epithelial cells, Tfr1 might be involved in the process of epithelial‐to‐mesenchymal transition (EMT), accompanied by an increase of stem cell markers and high proliferation of intestinal epithelial progenitors.^[^ ^21^ ^]^ Consistently, we also found significantly enriched EMT gene signature (FDR *q*‐value = 0.0024) and upregulated stem cell markers in our RNA‐seq analysis of *Tfr1‐*deficient iBAT. Nevertheless, these observed abnormal phenotypes could not be reversed by enforced iron overload, suggesting previously unrecognized role of Tfr1 gene itself rather than its iron uptake function.

In order to test whether iron deficiency plays a role in the transdifferentiation of adipocytes in the absence of Tfr1, we used both *Tmprss6* knockout and maternal low‐iron‐diet fed mice, both of which had extremely low iron levels in iBAT. However, both the gene expression pattern of myogenesis differed from *Tfr1^Adp/Adp^* mice, suggesting that an iron‐independent function of Tfr1 plays a role in determining the cell fate of brown preadipocytes, warranting further study.

## 4 Conclusion

Tfr1 plays a regulatory role in controlling both the thermogenic capacity and the development of beige/brown adipocytes via distinct mechanisms. During beigeing, *Tfr1* expression is transcriptionally regulated by HIF1α; in contrast, in iBAT, *Tfr1* deficiency facilitates the transdifferentiation of brown progenitor cells into either white adipocytes or muscle cells. The effect of *Tfr1* deficiency on beige fat could be attributed to iron deficiency due to impaired mitochondrial function, whereas the transdifferentiation of brown fat caused by *Tfr1* depletion is not due to iron deficiency. Taken together, these findings provide compelling evidence that Tfr1 plays an essential role in controlling both thermogenic capacity and the cell fate of thermogenic adipocytes.

## 5 Experimental Section

##### Animals

Breeding pairs of *C57BL/6J* mice (6–8 weeks old) were purchased from Shanghai SLRC Laboratory Animal Co., Ltd. or the animal facility of Guangzhou Chinese Medicine University, and housed under specific pathogen‐free (SPF) conditions in a 12‐h/12‐h light/dark cycle with free access to rodent chow and deionized water. *Adipoq‐Cre*, *Hif1α^fl/fl^*, and *Tfr1^fl/fl^* mice were kindly provided by Dr. Dahai Zhu (Institute of Basic Medical Sciences, Chinese Academy of Medical Sciences), Dr. Rong Ju (Zhongshan Ophthalmic Center, Sun Yat‐Sen University), and Dr. Ying Shen,^[^ ^39^ ^]^ respectively. *Ucp1‐Cre*, *Cmv‐Cre*, and *Gt(ROSA)26Sor^tm5(CAG‐Sun1/sfGFP)Nat^* mice were purchased from Jackson Laboratory (strain numbers 024670, 006054, and 021039, respectively). *Tmprss6^fl/fl^* mice were purchased from Shanghai Model Organisms. *Tfr1^Adp/Adp^*, *Tfr1^Ucp1/Ucp1^*, *Hif1α^Ucp1/Ucp1^*, and *Tmprss6^−/−^* mice were generated by crossing *Adipoq‐Cre*, *Ucp1‐Cre*, and *Cmv‐Cre* mice with mice carrying floxed *Tfr1*, *Hif1α*, and *Tmprss6* alleles, where appropriate. The primer sequences used for genotyping are listed in Table S1, Supporting Information. To induce thermogenesis and beigeing, the mice either received 7 daily intraperitoneal injections of CL‐316,243 (0.1 mg kg^−1^) or were exposed to cold (4 °C) for 12 h for the first 3 days, followed by 24 h cold‐treatment for additional 4 days. Core temperature was monitored every hour using a rectal thermometer (RWD Life Science, Shenzhen, China). Lean mass and fat mass were measured using low‐field NMR instrument (QMR06‐090H, Suzhou Niumag Analytical Instrument Corporation, China). All animal experiments were approved by the Institutional Animal Care and Use Committee of Zhejiang University or Guangdong Institute of Microbiology.

##### Metabolic Study

At 6–7 weeks of age, male *Tfr1^Adp/Adp^* mice and control (*Tfr1^fl/fl^*) littermates were fed a high‐fat diet (HFD; D12492, Research Diets, Inc., Brunswick, NJ) for at least 12 weeks. Serum total triglyceride (TG), total cholesterol (TC), low‐density lipoprotein‐cholesterol (LDL‐c), and non‐esterified fatty acid (NEFA) levels were then measured (Nanjing Jiancheng Bioengineering Institute, China) in accordance with the manufacturer's instructions. Glucose tolerance and insulin resistance were assessed using the glucose tolerance test (GTT) and insulin tolerance test (ITT), respectively, as previously described.^[^ ^40^ ^]^ After 12 weeks of consuming the HFD, whole‐body energy expenditure was monitored using indirect calorimetry with the Comprehensive Lab Animal Monitoring System (CLAMS, Columbus Instruments). Oxygen consumption (VO_2_), carbon dioxide production (VCO_2_), and energy expenditure were measured every 10 min.

##### qRT‐PCR

Total RNA was extracted from tissues and primary cells using TRIzol reagent (Thermo Fisher) in accordance with the manufacturer's instructions. RNA concentration was measured using a BioSpec‐nano (Shimadzu Corporation, Kyoto, Japan), and 1 µg total RNA was used for reverse transcription in a 20 µL reaction using the 5X All‐In‐One RT MasterMix (Applied Biological Materials, Richmond, Canada), followed by qRT‐PCR using the PowerUp SYBR Green Master Mix (Thermo Fisher) in a QuantStudio 6 Flex Real‐Time PCR System (Thermo Fisher). Each target gene's expression level was normalized to the expression of the respective internal control gene (*Rps18*, *Hprt*, or *Ppia*). The primers used for qRT‐PCR were listed in Table S2, Supporting Information.

##### Protein Isolation and Western Blot Analysis

Total proteins were isolated from adipose tissues by dissection and homogenization in a RIPA lysis buffer containing 150 mm NaCl, 1% NP‐40, 0.1% SDS, 25 mm Tris‐HCl (pH 7.4), 0.5% sodium deoxycholate, and 1X complete proteasome inhibitor cocktail. To isolate membrane proteins, adipose tissues were homogenized on ice in hypotonic buffer containing 25 mm NaCl, 25 mm Tris‐HCl (pH 7.4), and 1X complete proteasome inhibitor cocktail. The homogenate was then centrifuged at 16 000 *g* for 10 min, the supernatant was centrifuged at 41 000 *g* for 1 h, and the pellet (containing cellular membranes) was re‐suspended and sonicated. Total and membrane protein concentrations were measured using the Pierce BCA Protein Assay kit (Thermo Fisher), and 10 µg of total or membrane protein was resolved in a 10% SDS‐PAGE and transferred to a PVDF membrane. The following primary antibodies were used for immunoblotting: anti‐Ucp1 (ab10983, Abcam), anti‐PGC1α (AB3242, Millipore), anti‐Tfr1 (ab84036, Abcam), anti‐HIF1α (D123654, Sangon Biotech), anti‐Tubulin (D225847, Sangon Biotech), anti‐Cav1 (D161423, Sangon Biotech), anti‐PPARγ (A19676, Abclonal), anti‐HSL (4107, Cell Signaling Technology), anti‐p‐HSL (Ser563, 4139, Cell Signaling Technology), anti‐p‐HSL (Ser660, 4126, Cell Signaling Technology), and anti‐mitochondrial complex (ab110413, Abcam). Proteins were visualized using the ECL method (Thermo Fisher), and images were captured using the Bio‐Rad Imaging System (Bio‐Rad).

##### Serum and Tissue Non‐Heme Iron Assay

Serum iron was measured using the Iron/TIBC Reagent kit (I7506‐60; Pointe Scientific). Tissue non‐heme iron was measured using the standard chromogen method as described previously.^[^ ^41^ ^]^ In brief, homogenized animal tissues were incubated in NHI acid solution (10% trichloroacetic acid in 3 m HCl) at 65–70 °C for 72 h. After centrifugation to pellet the debris, 10 µL supernatant was loaded into each well in a 96‐well plate; 200 µL working solution containing 0.2% thioglycolic acid and 0.02% disodium‐4,7‐diphenyl‐1,10‐phenanthroline disulfonate in 50% saturated NaAc solution was added to each well, mixed, and the plate was incubated at room temperature for 10 min, after which the plate was read at 535 nm using a spectrophotometer plate reader (Thermo Fisher). Iron concentration was normalized to the wet tissue weight and is presented as µg g^−1^ wet tissue.

##### Paraffin Embedding, Sectioning, and H&E Staining

Adipose tissues were fixed in 10% formalin for 48 h. After dehydration, the fixed tissues were embedded in paraffin and sectioned at 4 µm. For staining, the sections were incubated at 65 °C for 30 min to melt the paraffin, followed by deparaffinization (3 rinses in xylene, 5 min each) and rehydration (100% alcohol for 2 min, followed by two rinses in 95% alcohol for 2 min each, 80% alcohol for 2 min, and pure water for 2 min). The sections were performed subsequently following the standard H&E protocol and mounted with DPX Mountant (Sigma‐Aldrich).

##### Micro‐PET/CT

The functional imaging of brown fat was performed as previously reported.^[^ ^42^ ^]^ In briefly, mice were acclimated to a cold environment at 4 °C for 4 hours or treated with 1 mg kg^−1^ CL‐316,243 i.p. for 1 h. The mice then received an intraperitoneal injection of ^18^F‐FDG and remained at 4 °C or RT for an additional 1 h. The mice were anesthetized and subject to CT and PET scans. ^18^F‐FDG uptake in iBAT was calculated as a standard uptake value (SUV), which is based on the ratio of the total signal within a volume of interest (VOI) to the injected dose and body weight.

##### Immunofluorescence

The tissue sections were deparaffinized and rehydrated as described above. After rehydration, antigen retrieval was performed using boiling antigen retrieval buffer (1.21 g Tris, 0.37 g EDTA, and 0.5 mL Tween 20 dissolved in 1 L ddH_2_O) for 1 h and then permeabilized in phosphate‐buffered saline (PBS) containing 0.5% Triton X‐100. The sections were then incubated with blocking buffer (PBS with 5% w/v BSA and 5% v/v normal goat serum) at room temperature for 1 h, followed by overnight incubation at 4 °C with the following primary antibodies: anti‐HIF1α (D123654, Sangon Biotech), anti‐Perilipin 1(D1D8, Cell Signaling), anti‐myosin H1E (MF 20, DSHB, Iowa City, IA), anti‐dystrophin (7A10, DSHB, Iowa City, IA), or anti‐myogenin (F5D, DSHB, Iowa City, IA). The sections were then incubated for 1 h at room temperature in the dark in the appropriate secondary antibodies (diluted in blocking buffer at 1:500). The nuclei were counterstained with DAPI‐containing mounting medium (F6057, Sigma).

##### RNA Sequencing and Bioinformatics Analysis

Total RNA was isolated from brown adipose tissue of *Tfr1^Adp/Adp^* and control littermates and sequenced using a BGI‐SEQ500 platform (Beijing Genomics Institute) or Illumina HiSeq X Ten platform (Biomarker Technologies Corporation, Beijing, China). Raw RNA‐seq reads in FASTQ format were passed through FASTQC for quality check, and low‐quality reads were removed using the FASTX‐Toolkit. The high‐quality reads were mapped to the mouse genome (GRCm38/mm10) using TopHat at option setting of ‐G mouse_mRNA.gtf and assembled against mouse mRNA annotation using HTSeq.^[^ ^43^ ^]^ Differential expression was analyzed and the fold change was calculated using the ratio of either CL‐316,243/saline or *Tfr1^Adp/Adp^*/*Tfr1^fl/fl^*, using DESeq2 package in R. Genes were considered to be significantly upregulated or downregulated at *p* < 0.05. Heatmaps were generated using the pheatmap package in R based on read counts of differentially expressed genes. Gene ontology (GO) analysis was performed using the R package cluster profiler for differentially expressed genes. The *p*‐values were corrected for multiple comparisons using the Benjamini‐Hochberg method. Differentially expressed genes (*p* < 0.05) were further analyzed using Gene Set Enrichment Analysis (GSEA). Both upregulated and downregulated genes were categorized with the Hallmark gene sets.

##### Chromatin Immunoprecipitation and High‐Throughput Sequencing and Bioinformatics Analysis

At 8 weeks of age, *GFP^Ucp1/Ucp1^* mice received five intraperitoneal injections of CL‐316,243 or were exposed to 4 °C to induce beigeing. The mice were then sacrificed, and the heart was perfused with 1% paraformaldehyde to crosslink histones or transcription factors with the chromatin, followed by quenching in glycine (125 mm). The GFP^+^ nuclei in beige adipocyte were isolated using flow cytometry (BD Biosciences, San Jose, CA). Chromatin in the nuclei of beige adipocytes was sheared at 4 °C using a Bioruptor Pico Sonicator (Diagenode) for 15 cycles with a 30 s on/off program. The sheared chromatin (≈250–400 bp) was then used for chromatin immunoprecipitation using a True MicroChIP Kit (C01010130, Diagenode) in accordance with the manufacturer's instructions. For immunoprecipitation, anti‐rabbit IgG (C15410206, Diagenode), anti‐HIF1α (C15410234, Diagenode), or anti‐H3K9/14Ac (C15410005, Diagenode) was mixed with the sheared chromatin DNA and incubated overnight at 4 °C. The precipitated DNA was then washed and reverse cross‐linked. The purified, precipitated chromatin DNA was then used to construct the sequencing library for either high‐throughput sequencing on a BGI‐SEQ500 platform or ChIP‐qPCR. For ChIP‐seq data analysis, the raw sequencing reads in FASTQ format were tested for quality control using FASTQC, followed by removal of low‐quality reads using the Fastx toolkit. The high‐quality reads were mapped to the mouse genome using Bowtie2.0 with one unique read. The ChIP‐seq enrichment peaks were called using MACS1.4.2 and annotated using Great 2.0.2. The primers used for ChIP‐qPCR were listed in Table S2, Supporting Information.

##### ITRAQ Labeling and Fractionation Using High‐pH Reverse‐Phase Chromatography

Vacuum‐dried tryptic peptides were labeled using 8‐plex iTRAQ reagents (AB SCIEX, Redwood City, CA) in accordance with the manufacturer's instructions. The reaction was quenched by incubation in ddH_2_O for 30 min and was concentrated and dried in a Savant DNA120 SpeedVac (Thermo Fisher Scientific), followed by the first dimensional fractionation on a high‐pH reverse chromatography column (ZORBAX Extended‐C18 2.1, Agilent Technologies) using a separation gradient with buffer B (10 mm ammonium dissolved in 90% acetonitrile, pH 10.0), which was increased linearly from 5% up to 30% over 40 min at a flow rate of 0.3 ml min^−1^. A total of 40 fractions were collected at even intervals. For every minute, four fractions were combined, and 15 fractions were finally obtained and used in the LC‐MS analysis.

##### Reverse‐Phase Nanoflow HPLC and Tandem Mass Spectrometry

Reverse‐phase nano‐LC‐MS/MS analysis was performed using the Eksigent nanoLC‐Ultra 2D System (AB SCIEX). The lyophilized fractions were suspended in 2% acetonitrile and 0.1% formic acid, and then loaded on a ChromXP C18 (3 µm, 120 Å) nanoLC trap column. Online trapping and desalting were performed at 2 µL min^−1^ for 10 min with 100% solvent A (98% water, 2% acetonitrile, and 0.1% formic acid). Next, an elution gradient of 5–40% acetonitrile (with 0.1% formic acid) over 70 min was used in a ChromXP analytical column (75 µm × 15 cm, C18, 3 µm, 120 Å, Eksigent). LC‐MS/MS analysis was then performed using a TripleTOF 5600 System (AB SCIEX) fitted with a NanoSpray III ion source (AB SCIEX). Data were acquired using an ion spray voltage of 2.4 kV, curtain gas at 30 PSI, nebulizer gas at 5 PSI, and an interface heater temperature of 150 °C. The MS stage was operated using TOF‐MS scans ranging from 400 to 1250 m z^−1^. For iminodiacetic acid, survey scans began at 100 to 1500 m z^−1^ and were acquired in 250‐ms intervals; as many as 30 product ion scans (80 ms) were collected if exceeding a threshold of 200 counts s^−1^ and with a +2 to +5 charge‐state. A Rolling collision energy setting was applied to all precursor ions for collision‐induced dissociation. Dynamic exclusion was set at one‐half of the peak width (≈16 s).

##### Protein Identification and Quantification

Using the MS/MS data, proteins were identified and quantified using ProteinPilot v.4.5 (AB SCIEX). The global false discovery rate was estimated using the integrated PSPEP tool in ProteinPilot to be 1.0% after searching against a decoy concatenated *Arachis hypogaea* Linn protein database containing 38 967 entries. The database search parameters were set as follows: iTRAQ 8‐plex peptide‐labeling quantification, cysteine modified with iodoacetamide, trypsin digestion, MS and MS/MS tolerance of 20 ppm and 0.1 Da, respectively, and thorough searching mode with minimum threshold of 95% confidence (unused protein score >1.3) at the protein level. For a protein to considered differentially expressed with a fold change >1.5 or <0.67, a minimum of two unique peptides were required and the *p*‐value was <0.05.

##### Transmission Electron Microscopy

Samples of brown or white adipose tissue (1 mm × 2 mm × 2 mm) were quickly harvested from the mouse and immediately fixed in 3% phosphate‐glutaraldehyde. The Electron Microscopy Core Facility of Zhejiang University post‐fixed, embedded, cut, and mounted the samples. The samples were then examined under a Tecnai 10 (100 kv) transmission electron microscope (FEI). At least three random views were recorded for each sample.

##### Primary Cell Isolation and Flow Cytometry Analysis

The stromal vascular fraction (SVF) of iBAT and iWAT were isolated as previously described.^[^ ^44^ ^]^ In brief, the adipose tissue was minced and then incubated in working solution (1.5 mg mL^−1^ collagenase type II, 2.4 U mL^−1^ dispase II, and 10 mm CaCl_2_) for 30 min at 37 °C. The cell suspension was then filtered and centrifuged. The suspended SVF cells were then incubated in erythrocyte‐lysing buffer for 5 min, spun down by centrifugation, and re‐suspended in PBS containing 5% fetal bovine serum (FBS). The following antibodies (at 1:100) were used to analyze the macrophages (all from Biolegend): anti‐CD11b (M1/70), anti‐F4/80 (BM8), and anti‐CD11c (N418). For analyzing stem cells, anti‐CD34 antibody (Biolegend, RAM34) was used. Labeled cells were analyzed using an ACEA NovoCyte flow cytometer (BD Biosciences). FACS data were analyzed using FlowJo (Tree Star, Inc., Ashland, OR). The gating map for analyzing macrophages is shown in Figure S3G, Supporting Information. The ratio of macrophages to total cells and inflammatory CD11c^+^ cells to total macrophages were calculated.

##### Cell Culture

Isolated SVF cells were re‐suspended in complete Dulbecco's modified Eagle's medium (DMEM) and seeded in collagen‐coated 6‐well or 12‐well plates; adipogenic differentiation was induced upon reaching 100% confluence. Primary cells were grown in induction medium (DMEM containing 10% FBS, 0.5 mm isobutylmethylxanthine, 125 nm indomethacin, 1 µm dexamethasone, 0.5 µm rosiglitazone, 20 nm insulin, and 1 nm T3) for 2–3 days before switching to differentiation medium (DMEM containing 10% FBS, 1 µm rosiglitazone, 20 nm insulin, and 1 nm T3). C3H10T1/2 cells were purchased from Cell Resource Center, Shanghai Academy of Life Sciences, Chinese Academy of Sciences and grown at 37 °C in 5% CO_2_ in DMEM containing 10% FBS, 100 UI mL^−1^ penicillin, and 100 µg mL^−1^ streptomycin.

##### Lentivirus Vector Construction, Packaging, and Infection

Two separate *Tfr1* shRNA constructs were cloned into the pLKO.1‐puro vector, which was used for lentivirus packaging. The sequences targeting the *Tfr1* mRNA were as follows: 5′‐AGACCGTTATGTTGTAGTACT‐3′ (sh*Tfr1*‐1) and 5′‐GACAATAACATGAAGGCTACT‐3′ (sh*Tfr1*‐2). The plasmid and the packaging vectors pCMV‐VSV‐G and psPAX2 were co‐transfected into HEK293T cells at 70% confluence, and the virus‐containing supernatant was collected 48 h later. The cells to be infected were incubated with the virus‐containing supernatant with 8 µg mL^−1^ polybrene for 48 h and then selected with 1.2 µg mL^−1^ puromycin. *Tfr1* mRNA and protein levels were measured in order to confirm knockdown efficiency.

##### Statistical Analysis

Summary data were presented as the mean ± SD. Data were analyzed using the unpaired Student's *t*‐test or one‐way ANOVA followed by a Bonferroni post hoc analysis, where appropriate. Differences with a *p*‐value < 0.05 were considered statistically significant. All statistical analyses were performed using Prism (GraphPad, San Diego, CA). Representation of the *p‐value* was *p < 0.05, **p < 0.01, ***p < 0.001, and N.S.: not significant (*p* > 0.05).

## Conflict of Interest

The authors declare no conflict of interest.

## Supporting information

Supporting Information
