# Supplementary material for: Transferrin Receptor 1 Regulates Thermogenic Capacity and Cell Fate in Brown/Beige Adipocytes
Source: Adv Sci (Weinh). 2020 Apr 24;7(12):1903366. doi: 10.1002/advs.201903366 (PMC7312276; doi:10.1002/advs.201903366)
Supplement: Supplementary file 1 — Supporting Information [file ADVS-7-1903366-s001.pdf]

Copyright WILEY-VCH Verlag GmbH & Co. KGaA, 69469 Weinheim, Germany, 2020.

ADVANCED SCIENCE
Open Access

Supporting Information

for *Adv. Sci.,* DOI: 10.1002/advs.201903366

Transferrin Receptor 1 Regulates Thermogenic Capacity and Cell Fate in Brown/Beige Adipocytes

Jin Li, Xiaohan Pan, Guihua Pan, Zijun Song, Yao He, Susu Zhang, Xueru Ye, Xiang Yang, Enjun Xie, Xinhui Wang, Xudong Mai, Xiangju Yin, Biyao Tang, Xuan Shu, Pengyu Chen, Xiaoshuang Dai, Ye Tian, Liheng Yao, Mulan Han, Guohuan Xu, Huijie Zhang, Jia Sun, Hong Chen, Fudi Wang, Junxia Min,* and Liwei Xie**

WILEY-VCH

Copyright WILEY-VCH Verlag GmbH & Co. KGaA, 69469 Weinheim, Germany, 2020.

Supporting Information

Transferrin Receptor 1 Regulates Thermogenic Capacity and Cell Fate in Brown/Beige Adipocytes

Jin Li, Xiaohan Pan, Guihua Pan, Zijun Song, Yao He, Susu Zhang, Xueru Ye, Xiang Yang, Enjun Xie, Xinhui Wang, Xudong Mai, Xiangju Yin, Biyao Tang, Xuan Shu, Pengyu Chen, Xiaoshuang Dai, Ye Tian, Liheng Yao, Mulan Han, Guohuan Xu, Huijie Zhang, Jia Sun, Hong Chen, Fudi Wang, Junxia Min* and Liwei Xie**

Figure and Figure legends

Figure S1

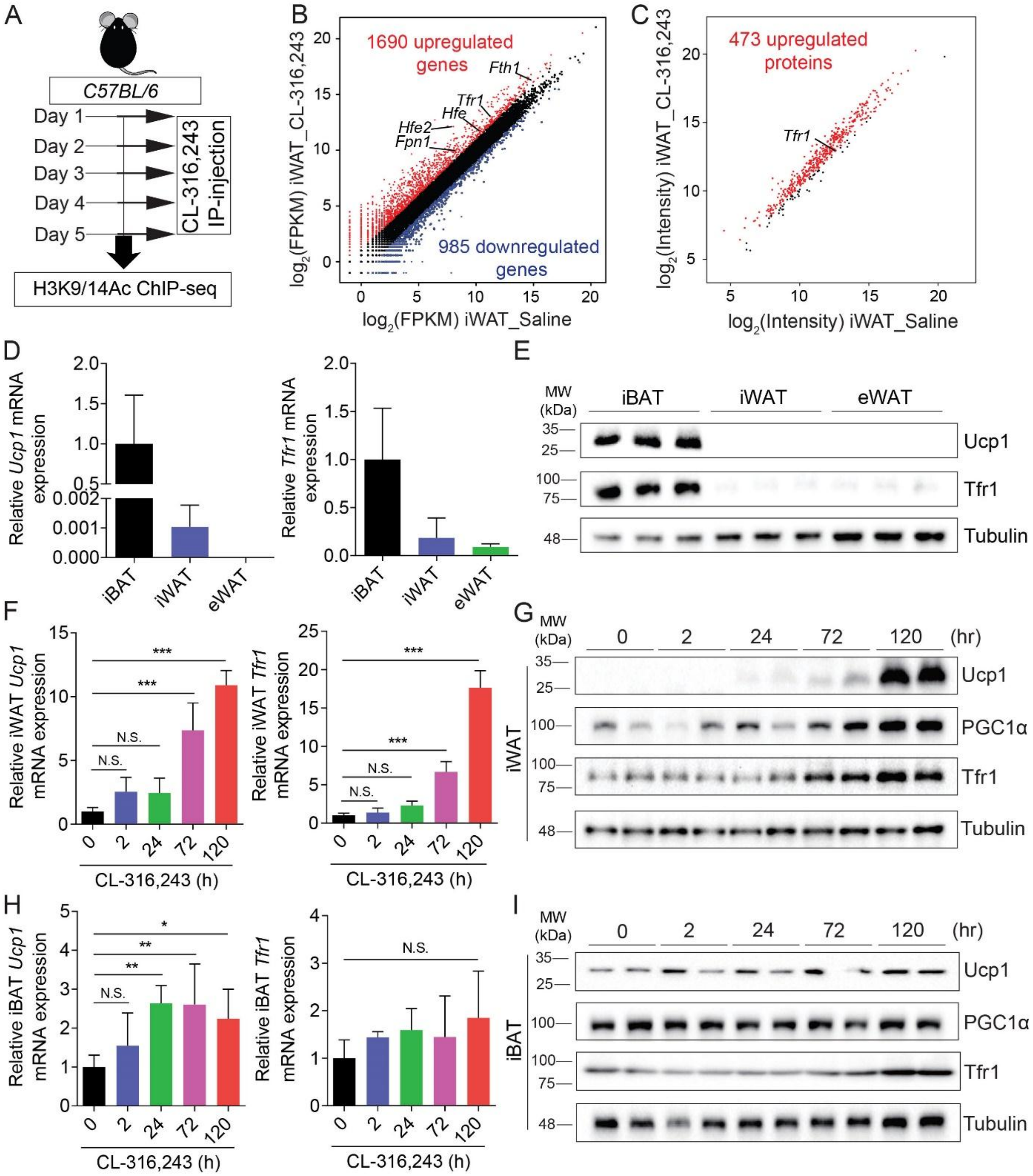

Figure S1. Related to Figure 1. CL-316,243 increases Tfr1 expression primarily in beige adipocytes. (**A**) Timeline for CL-316,243 injections to induce beigeing of white adipocyte in C57BL/6J mice and

H3K9/14Ac ChIP-seq analysis. (**B**) Scatter plot of differentially expressed genes in iWAT following CL-316,243 injections. (**C**) Scatter plot of membrane-enriched proteins following CL-316,243 injections. (**D and E**) *Ucp1* and *Tfr1* mRNA and protein measured in iBAT, iWAT, and eWAT of *C57BL/6J* mice at steady state (n=6-8 mice/group), presented as mean ± SD, pooled from 3 independent experiments. (**F and H**) qRT-PCR analysis of *Ucp1* and *Tfr1* mRNA in iWAT and iBAT of *C57BL/6J* mice 0, 2, 24, 72, and 120 hr after CL-316,243 injections (n=6 mice/group), presented as mean ± SD, pooled from 3 independent experiments. (**G and I**) Ucp1, PGC1α, Tfr1, and Tubulin protein in iWAT and iBAT of *C57BL/6J* mice 0, 2, 24, 72, and 120 hr after CL-316,243 injections (n=6 mice/group), presented as mean ± SD, pooled from 3 independent experiments. *P<0.05, **P<0.01, ***P<0.001, and N.S., not significant. One-way ANOVA with a Bonferroni post-hoc analysis was used for comparison among multiple groups.

Figure S2

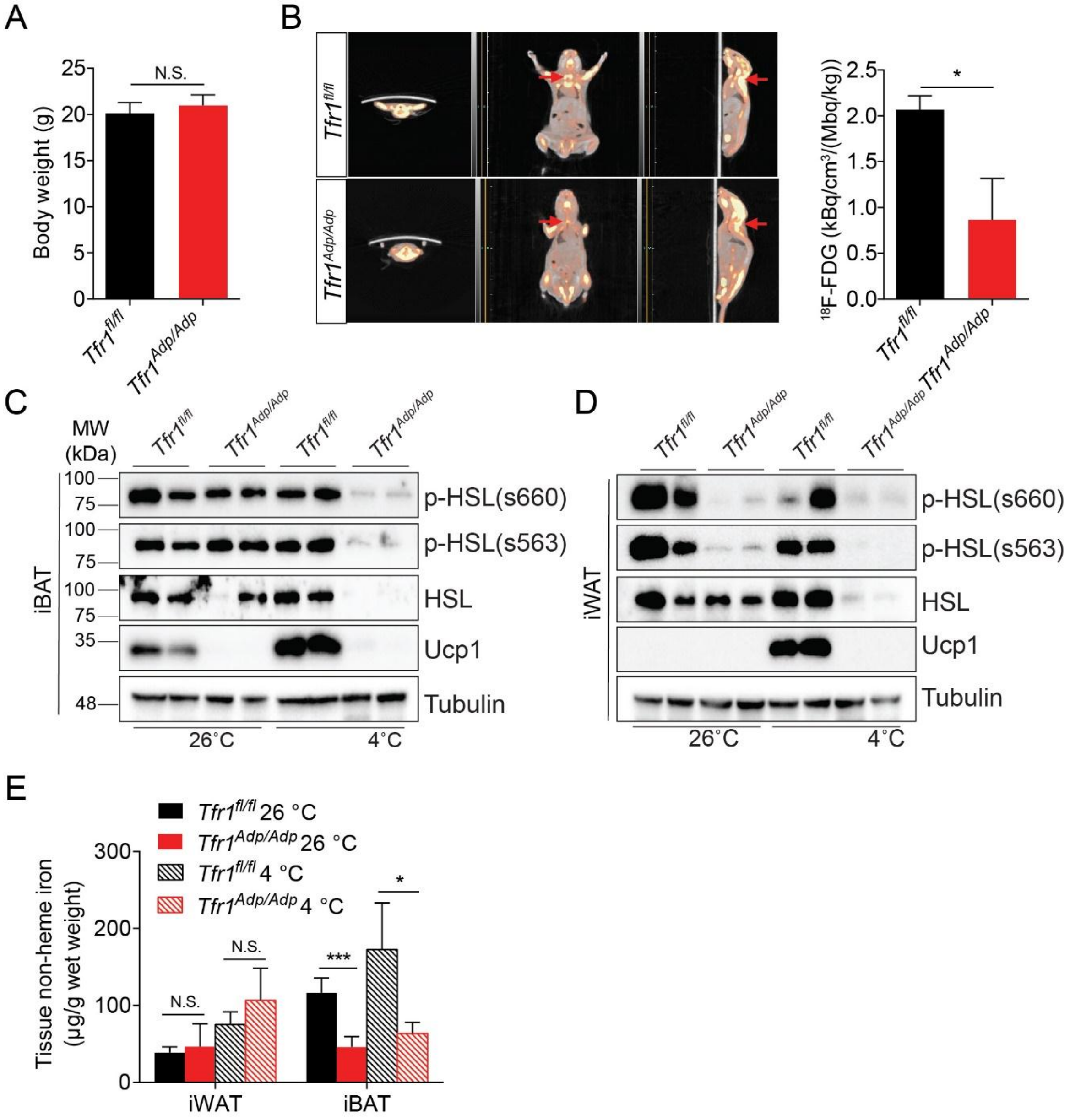

Figure S2. Related to Figure 2. Metabolic phenotype of control and *Tfr1*$^{Adp/Adp}$ mice. (**A**) Body weight of 8-week-old control (*Tfr1*$^{fl/fl}$) and *Tfr1*$^{Adp/Adp}$ mice (n=4-5 mice/group), presented as mean ± SD, pooled from 2 independent experiments. (**B**) Example ^{18}F-FDG micro-PET/CT images of *Tfr1*$^{fl/fl}$ and *Tfr1*$^{Adp/Adp}$ mice after exposure to cold. The quantitative summary of FDG uptake is shown at the right (n=3 mice/group), presented as mean ± SD. (**C**) Representative western blot analysis of HSL, pHSL(s660), p-HSL(s563), Ucp1, and Tubulin proteins in iBAT of *Tfr1*$^{fl/fl}$ and *Tfr1*$^{Adp/Adp}$ housed at 26°C

or 4°C (n=4 mice/group). (**D**) Representative western blot analysis of HSL, pHSL(s660), p-HSL(s563), Ucp1, and Tubulin proteins in iWAT of *Tfr1*$^{fl/fl}$ and *Tfr1*$^{Adp/Adp}$ housed at 26°C or 4°C (n=4 mice/group). (**E**) The non-heme iron levels were detected in iBAT and iWAT of *Tfr1*$^{fl/fl}$ and *Tfr1*$^{Adp/Adp}$ mice housed for 7 days at 26°C or 4°C (n=4-6 mice/group), presented as mean ± SD, pooled from 2 independent experiments. *$P<0.05$, ***$P<0.001$, and N.S., not significant. Unpaired Student's *t*-test was used for comparison between two groups. One-way ANOVA with a Bonferroni post-hoc analysis was used for comparison among multiple groups.

Figure S3

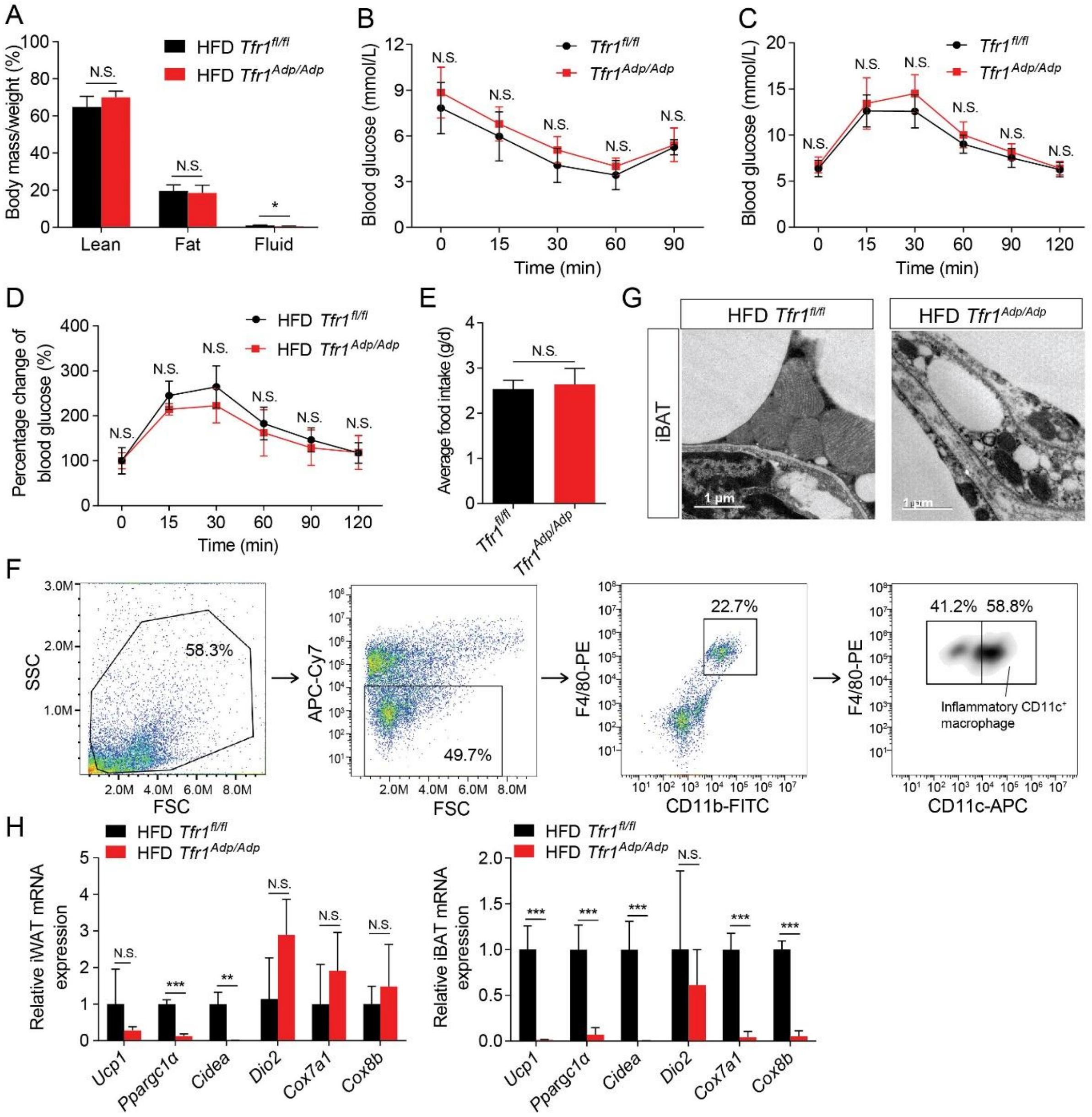

Figure S3. Related to Figure 3. Metabolic phenotype of HFD-fed control and *Tfr1*$^{Adp/Adp}$ mice. (**A**) Quantification of lean mass, fat mass and fluid of HFD-fed *Tfr1*$^{Adp/Adp}$ and control mice by Low-field NMR instrument (QMR06-090H, Suzhou Niumag Analytical Instrument Corporation, China) (n=4-6 mice/group), presented as mean ± SD**.** (**B and C**) Blood glucose was measured during the glucose tolerance test (GTT, B) and insulin tolerance test (ITT, C) in 2-month-old *Tfr1*$^{fl/fl}$ and *Tfr1*$^{Adp/Adp}$ mice (n=7 mice/group), presented as mean ± SD, pooled from 2 independent experiments. (**D**) The

percentage change of blood glucose of HFD-fed *Tfr1*$^{Adp/Adp}$ and control mice in GTT (n=7-8 mice/group). presented as mean ± SD, pooled from 2 independent experiments. (**E**) Average food intake of HFD-fed *Tfr1*$^{Adp/Adp}$ and control mice were measured for 5 days (n=6 mice/group), presented as mean ± SD, pooled from 2 independent experiments. (**F**) Flow cytometry results of antibody labeling and gating. FSC, forward scatter; SSC, side scatter. (**G**) Representative transmission electron microscopy images of iBAT in an HFD-fed *Tfr1*$^{fl/fl}$ mouse and an HFD-fed *Tfr1*$^{Adp/Adp}$ mouse (n=3 mice/group). (**H**) qRT-PCR analysis of *Ucp1, Ppargc1α, Cidea, Dio2, Cox7a1* and *Cox8b* mRNA in iWAT (left) and iBAT (right) of HFD-fed *Tfr1*$^{Adp/Adp}$ and control mice (n=3-4 mice/group), presented as mean ± SD, pooled from 2 independent experiments. **P*<0.05, ***P<0.001, and N.S., not significant. Unpaired Student's *t*-test was used for comparison between two groups. One-way ANOVA with a Bonferroni post-hoc analysis was used for comparison among multiple groups.

Figure S4

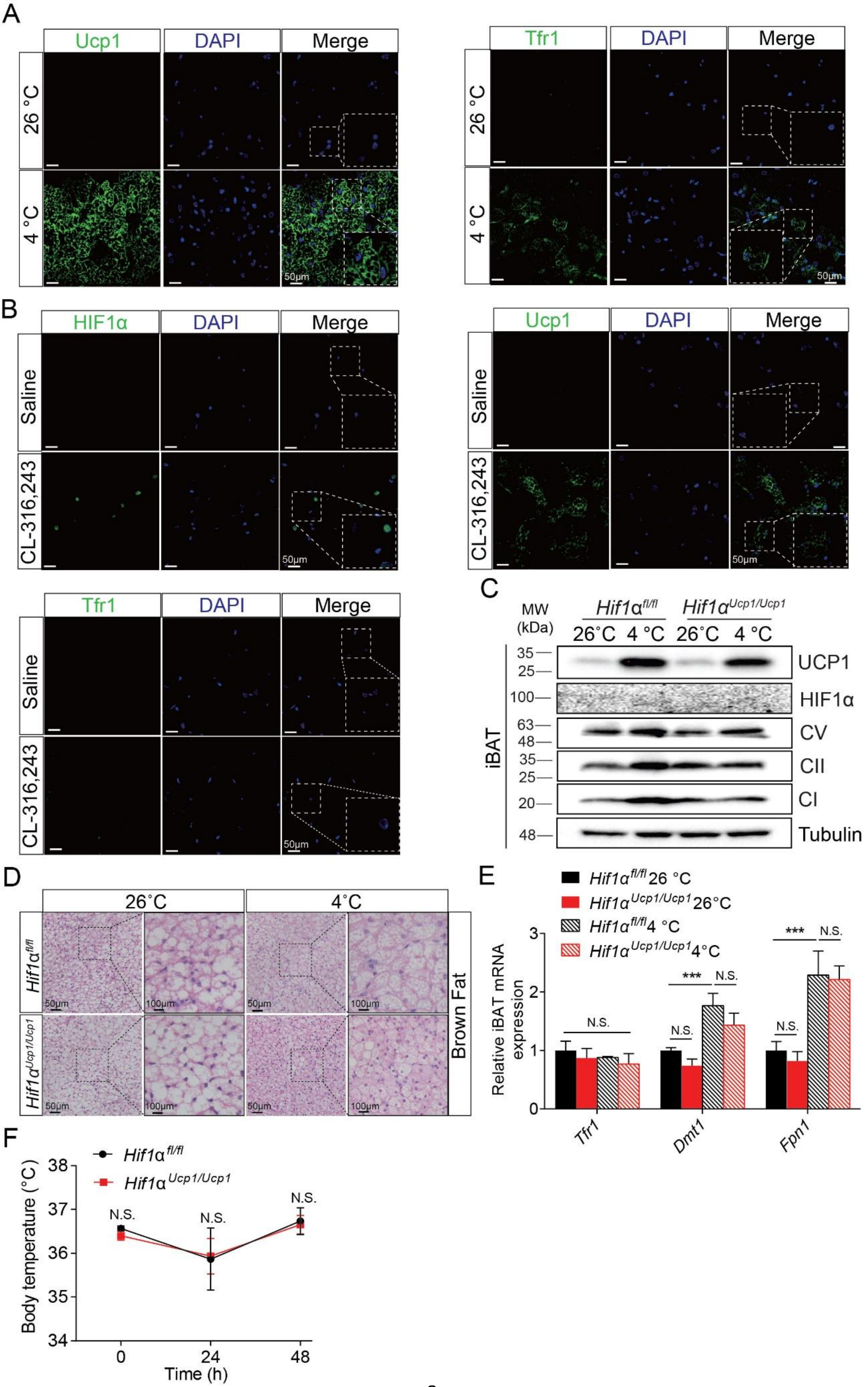
A
Ucp1
DAPI
Merge
26 °C
4 °C
50μm
Tfr1
DAPI
Merge
B
HIF1α
Saline
CL-316,243
Ucp1
Tfr1
C
MW (kDa)
$Hif1\alpha^{fl/fl}$
$Hif1\alpha^{Ucp1/Ucp1}$
26°C
4 °C
iBAT
UCP1
HIF1α
CV
CII
CI
Tubulin
D
26°C
4°C
Brown Fat
100μm
E
$Hif1\alpha^{fl/fl}$ 26 °C
$Hif1\alpha^{Ucp1/Ucp1}$ 26°C
$Hif1\alpha^{fl/fl}$ 4 °C
$Hif1\alpha^{Ucp1/Ucp1}$ 4°C
Relative iBAT mRNA expression
N.S.
***
Tfr1
Dmt1
Fpn1
F
Body temperature (°C)
Time (h)

Figure S4. Related to Figure 4. Loss of Hif1α in iBAT does not cause impaired thermogenesis. (**A**) Representative images of white adipocytes obtained from C57BL/6J mice housed at 26°C or 4°C and immunostained for Tfr1 (left) or Ucp1 (right); the nuclei were counterstained with DAPI (n=6 mice/group). (**B**) Representative images of white adipocytes obtained from control-treated (saline) and CL-316,243–treated C57BL/6J mice and immunostained for HIF1α (top left), Tfr1 (top right), or Ucp1 (bottom left); the nuclei were counterstained with DAPI (n=6 mice/group). (**C**) Representative western blot analysis of Ucp1, HIF1α, Tubulin, and mitochondrial complex I, II, and V proteins in iBAT of *Hif1*$\alpha^{fl/fl}$ *and Hif1*$\alpha^{Ucp1/Ucp1}$ mice housed at 26°C or 4°C (n=4 mice/group). (**D**) Representative images of H&E-stained iBAT sections from *Hif1*$\alpha^{fl/fl}$ *and Hif1*$\alpha^{Ucp1/Ucp1}$ mice housed at 26°C or 4°C (n=4 mice/group). (**E**) qRT-PCR analysis of *Tfr1*, *Dmt1*, and *Fpn1* expression in iBAT of *Hif1*$\alpha^{fl/fl}$ and *Hif1*$\alpha^{Ucp1Ucp1}$ mice housed at 26°C or 4°C for 7 days (n=4 mice/group), presented as mean ± SD, pooled from 2 independent experiments. (**F**) Time course of rectal temperature of *Hif1*$\alpha^{fl/fl}$ *and Hif1*$\alpha^{Ucp1/Ucp1}$ mice mice at 4°C (n=3 mice/group), presented as mean ± SD.****P*<0.001, and N.S., not significant. One-way ANOVA with a Bonferroni post-hoc analysis was used for comparison among multiple groups.

Figure S5

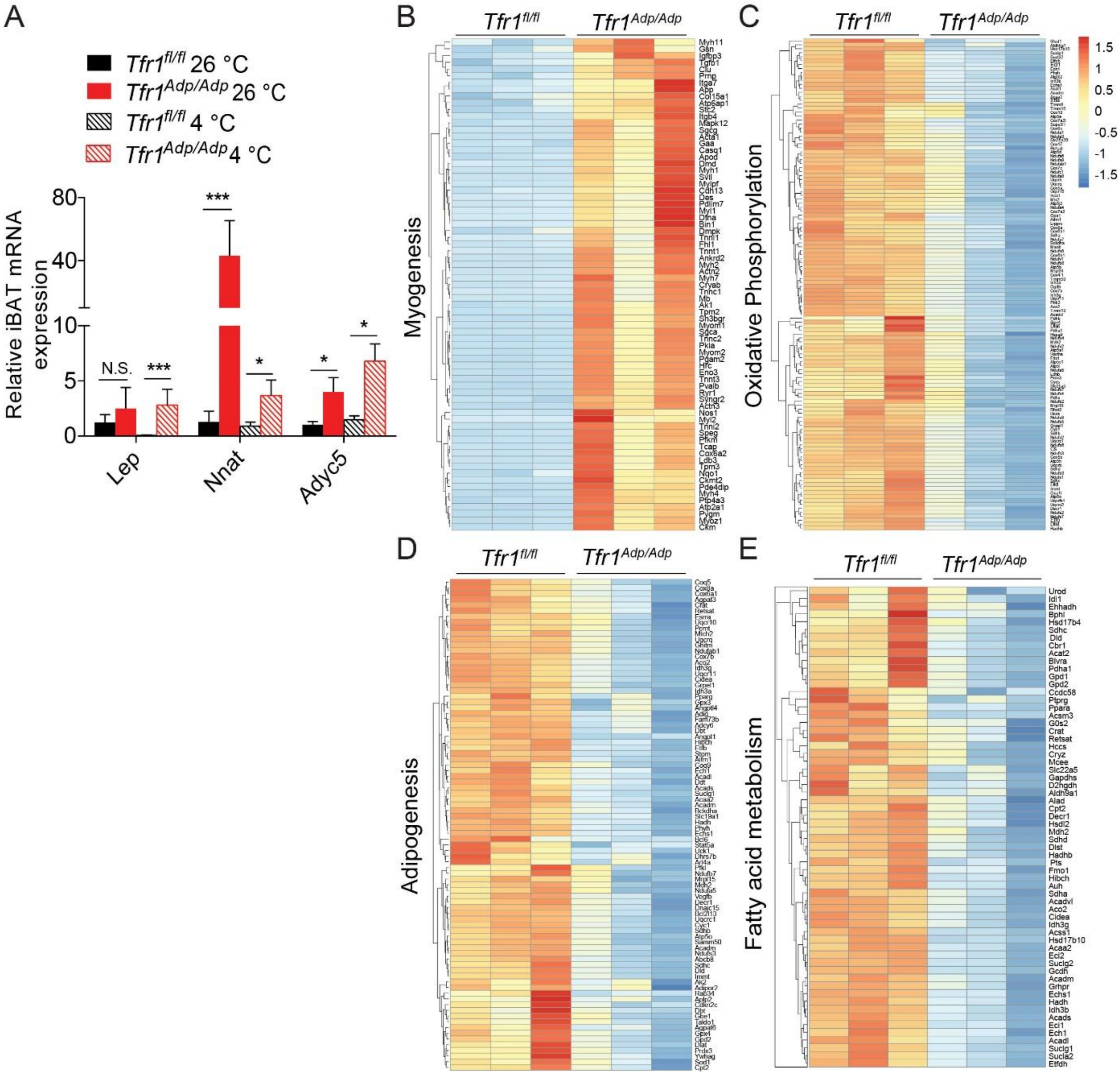

Figure S5. Related to Figure 5. White adipocyte marker genes are differentially expressed between *Tfr1*$^{Adp/Adp}$ and *Tfr1*$^{fl/fl}$ mice. (**A**) qRT-PCR analysis of the white adipocyte–related genes *Lep*, *Nnat*, and *Adcy5* in iBAT of *Tfr1*$^{fl/fl}$ *and Tfr1*$^{Adp/Adp}$ mice housed for 7 days at 26°C or 4°C n=6-8 mice/group, presented as mean ± SD, pooled from 2 independent experiments. (**B-E**) Heatmaps of differentially expressed genes associated with myogenesis, oxidative phosphorylation, adipogenesis, and fatty acid metabolism in iBAT of *Tfr1*$^{fl/fl}$ and *Tfr1*$^{Adp/Adp}$ mice; *$P<0.05$, ***$P<0.001$, and N.S., not significant.

One-way ANOVA with a Bonferroni post-hoc analysis was used for comparison among multiple groups.

Figure S6

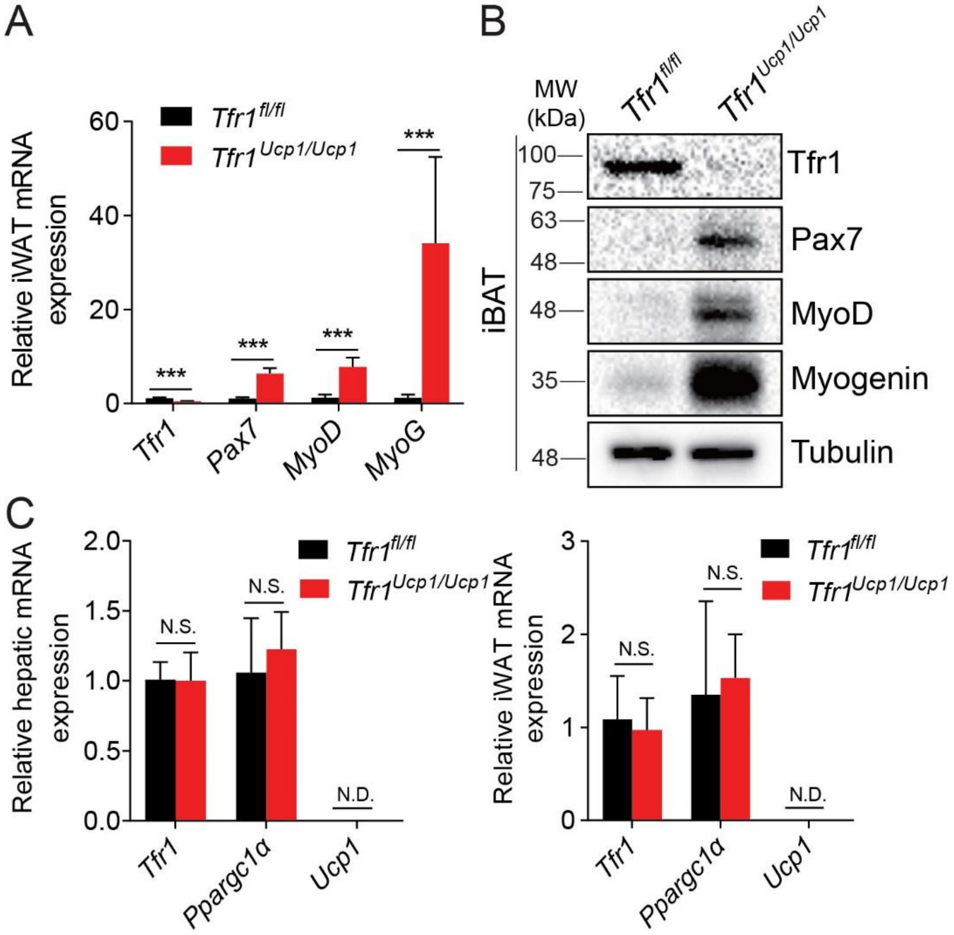

Figure S6. Related to Figure 5. Validation of thermogenic cell–specific *Tfr1* knockout and muscle cell marker gene expression. (**A**) qRT-PCR analysis of skeletal muscle–related gene expression in iBAT of *Tfr1*$^{fl/fl}$ and *Tfr1*$^{Ucp1/Ucp1}$ mice (n=6 mice/group), presented as mean ± SD, pooled from 2 independent experiments. (**B**) Representative western blot analysis of Tfr1, Pax7, MyoD, Myogenin, and Tubulin protein in iBAT of *Tfr1*$^{fl/fl}$ and *Tfr1*$^{Ucp1/Ucp1}$ mice (n=6 mice/group). (**C**) qRT-PCR analysis of *Tfr1*, *Ppargc1*α, and *Ucp1* mRNA in the liver (left) and iWAT (right) of *Tfr1*$^{fl/fl}$ and *Tfr1*$^{Ucp1/Ucp1}$ mice

(n=6 mice/group), presented as mean ± SD. ***P<0.001, N.S., not significant, and N.D., not detectable. Unpaired Student's *t*-test was used for comparison between two groups.

Figure S7

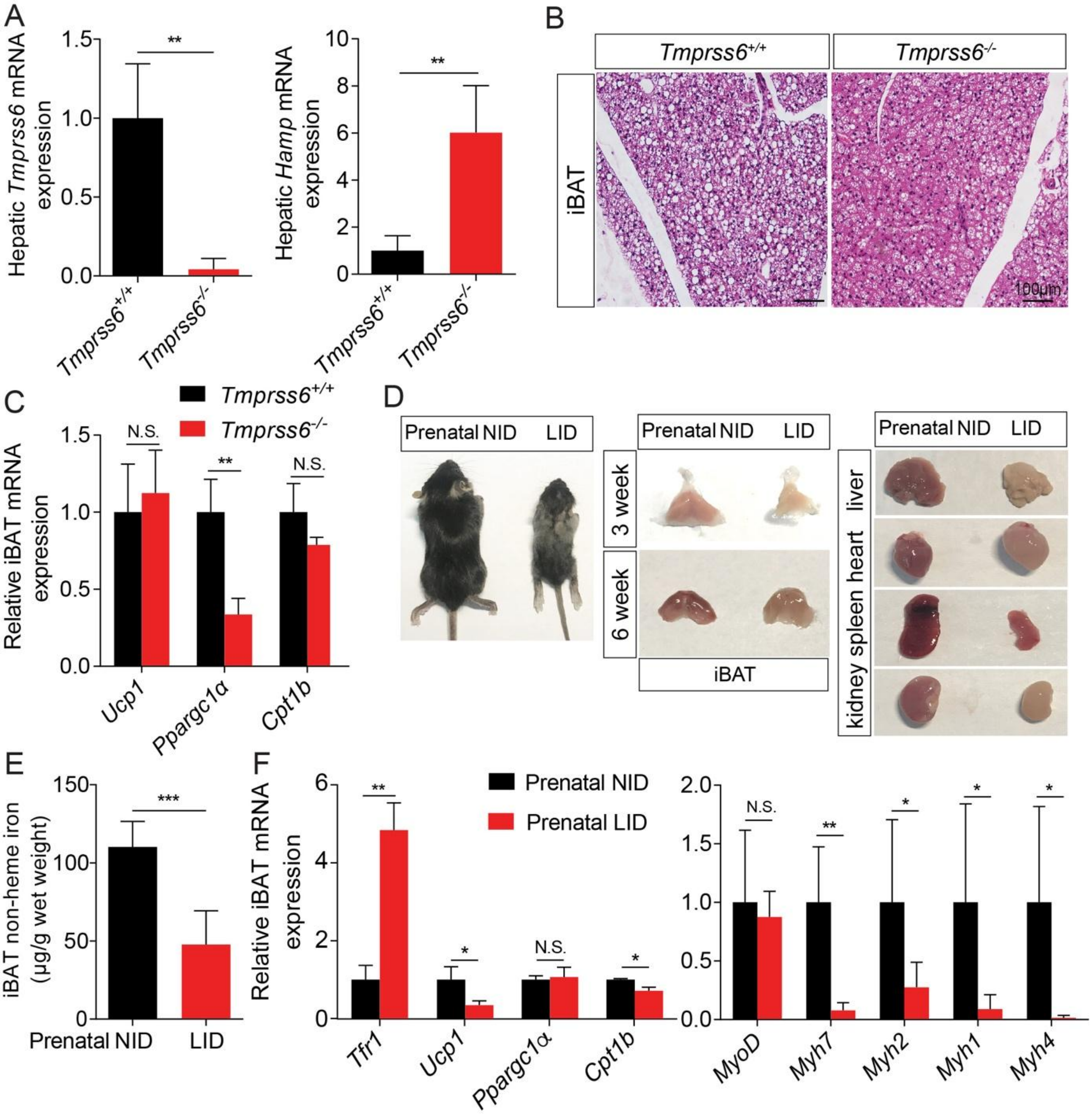

Figure S7. Related to Figure 6. Characterization of iron-deficient *Tmprss6* knockout mice and offspring exposed *in utero* and postnatally to a maternal low-iron diet. (**A**) qRT-PCR analysis of hepatic *Tmprss6* and *Hamp* mRNA in liver in control (*Tmprss6*$^{+/+}$) and *Tmprss6*$^{-/-}$ mice (n=4-6 mice/group), presented as mean ± SD, pooled from 2 independent experiments. (**B**) Representative images of H&E-stained iBAT from *Tmprss6*$^{+/+}$ and *Tmprss6*$^{-/-}$ mice (n=3 mice/group). (**C**) Representative images of iBAT, heart, liver, spleen, and kidney obtained from 3-week-old and 6-

week-old C57BL/6J mice that were exposed to either a normal-iron diet (NID) or a low-iron diet (LID) since conception and then maintained on that diet (n=6 mice/group), presented as mean ± SD, pooled from 2 independent experiments. (**D**) Representative images of H&E-stained iBAT in prenatal NID-fed and LID-fed mice (n=3 mice/group). (**E**) Non-heme iron level was measured in iBAT of prenatal NID-fed and LID-fed mice (n=6 mice/group), presented as mean ± SD, pooled from 2 independent experiments. (**F**) qRT-PCR analysis of thermogenic (left) and myogenic (right) genes in iBAT of prenatal NID-fed and LID-fed mice (n=6 mice/group), presented as mean ± SD, pooled from 2 independent experiments *P<0.05, **P<0.01, ***P<0.001, and N.S., not significant. Unpaired Student's t-test was used for comparison between two groups.

Table S1. Primers used for genotyping the knockout mice used in this study

**Knockout mouse line**	**Forward (5'→3')**	**Reverse (5'→3')**
Tfr1$^{fl/fl}$	CAGTAATCCCAGAGGAATCATTAG	CTAAACCGGGTGTATGACAATG
Hif1$\alpha^{fl/fl}$	TGCATGTGTATGGGTGTTTTG	GAAAACTGTCTGTAACTTCATTTCC
Tmprss6$^{fl/fl}$	CAAGGATCCCCTCAAACCCC	CACCTGGCTTACGGTCACTT
Ucp1-Cre	TGCTGTTTCACTGGTTATGCGG	TTGCCCCTGTTTCACTATCCAG
Adipoq-Cre	GGATGTGCCATGTGAGTCTG	ACGGACAGAAGCATTTTCCA
Cmv-Cre	ATTTGCCTGCATTACCGGTC	ATCAACGTTTTCTTTTCGG

Table S2. Primers used for qRT-PCR and ChIP-qPCR

	**Stem cell markers**	
**Gene**	**Forward (5'→3')**	**Reverse (5'→3')**
Dclk1	TGAGCATCCCTGGGTTAATGAT	GAAACTCCTGCTGCAGTGC
Cd44	TTCGATGGACCGGTTACCATAA	AGCTTTCTGGGGTGCTCTT
Ly6a	TCAATTACCTGCCCCTACCC	CAGAGGTCTTCCTGGCAACA
Cd34	CCAGGGTATCTGCCTGGAAC	TCAGCCTCCTCCTTTTCACA
Kit	AGAGATTTGGCAGCCAGGA	TCTCTGGTGCCATCCACTTC
EN1	ACAGCAACCCCTAGTGTGG	TAGCGGTTTGCCTGGAACT
	**Skeletal muscle markers**	
**Gene**	**Forward (5'→3')**	**Reverse (5'→3')**
Pax7	TGCCCTCAGTGAGTTCGATT	GAGGTCGGGTTCTGATTCCA
MyoD	CGCCACTCCGGGACATAG	GAAGTCGTCTGCTGTCTCAAAGG
MyoG	ATCCAGTACATTGAGCGCCT	GCAAATGATCTCCTGGGTTGG
Mstn	CCAGGACCAGGAGAAGATGG	AGTCCCATCCAAAGGCTTCA
Myh7(MYH I)	GCTGCAGCAGTTCTTCAACC	GGAACATGCACTCCTCCTCA
Myh2(MYH_IIA)	CCAAGGCCATGTATGAGAAGATGTT	AGCTGTTCCAGGCTGTTGA
Myh1(MYH_IIX)	CGCTGGCTTTGAGATCTTTGATT	CAGGTCCATCCCAAAGTCAATG

Myh4(MYH_IIB)	CCCTGGCCAAGTCCATGTAT	ACAGCTGCTCCAAGGTGT

Iron metabolism

**Gene**	**Forward (5'→3')**	**Reverse (5'→3')**
Dmt1	CCAGGATGTGGAGCACCTA	GCTTGTGAACGTGAGGATGG
Fpn1	TTGTGGCAGGAGAAAACAGG	GCCAATGACTGGAGAACCAA
Tfr1	TCGTACAGCAGCGGAAGT	TCTCCACGAGCGGAATACAG
Tmprss6	CATGGCTCCAGGCCATTGAT	GGGCATCCTTCAAGAGTGGAA
Hif1a	CAGAATGCTCAGAGGAAGCG	CTGCATGCTAAATCGGAGGG

Thermogenic genes

**Gene**	**Forward (5'→3')**	**Reverse (5'→3')**
Ppargc1a	CTCTGGAACTGCAGGCCTAA	TGCCTTGGGTACCAGAACA
Ucp1	ATACTGGCAGATGACGTCCC	CGAGTCGCAGAAAAGAAGCC
Cidea	ATACATCCAGCTCGCCCTTT	ACTTACTACCCGGTGTCCAT
Dio2	CAGTGTGGTGCACGTCTC	TGAACCAAAGTTGACCACCAG
Cox7a1	AGCTGCTGAGGACGCA	GCTTCTGCTTCTCTGCCAC
Cox8b	TTCCCAAAGCCCATGTCTCT	GGCTAAGACCCATCCTGCT
Cpt1b	GAATCCTCGACGACCCTTCC	TGAAGAAGGTCTGACGTGCC

White adipocyte markers

Gene	Forward (5'→3')	Reverse (5'→3')
Lep	GTGTCGGTTCCTGTGGCT	AGCCCAGGAATGAAGTCCAA
Nnat	TGCTGCAGACCCTGTCC	GCGTTGGTCGCGATCG
Adcy5	TCTTCGTGCTGGCTCTGT	CTGCAGCTCCTCCATCTCC
	**ChIP-qPCR**	
**Gene**	**Forward (5'→3')**	**Reverse (5'→3')**
Tfr1_HRE	TACTCCGCGCACGCACTGG	CGGCCCTCTGGATCTACG
Cav1_HRE	CCTTGGGGATGTGCCTAGA	AGGGGTTTGTTCTGCTCTCA
Pdk3_HRE	CCGCGACACCTACACAAG	TACCGGGGCTTTAAGGAAGC
	**H3K9Ac14 binding site**	
**Gene**	**Forward (5'→3')**	**Reverse (5'→3')**
Int_Ctrl_Chr5	CCCGTCACTCAACCATTTCA	CTTATCAATGGGGGCTCTGG
Tfr1	CTCCAAAGACACGGCGG	TAGAAAAGGCGCCAAGGC
Dmt1	CTGATCCGGAGGCGTGAT	GGGGATGGGAGTTGCCA
Fpn1	AGCGGCTTATAGGGAGCC	CGGACCTGGACGTCCAG
